# RiboRid: A low cost, advanced, and ultra-efficient method to remove ribosomal RNA for bacterial transcriptomics

**DOI:** 10.1371/journal.pgen.1009821

**Published:** 2021-09-27

**Authors:** Donghui Choe, Richard Szubin, Saugat Poudel, Anand Sastry, Yoseb Song, Yongjae Lee, Suhyung Cho, Bernhard Palsson, Byung-Kwan Cho

**Affiliations:** 1 Department of Biological Sciences, Korea Advanced Institute of Science and Technology, Daejeon, Republic of Korea; 2 Innovative Biomaterials Research Center, KI for the BioCentury, Korea Advanced Institute of Science and Technology, Daejeon, Republic of Korea; 3 Department of Bioengineering, University of California San Diego, La Jolla, California, United States of America; 4 Department of Pediatrics, University of California San Diego, La Jolla, California, United States of America; Chinese Academy of Sciences, CHINA

## Abstract

RNA sequencing techniques have enabled the systematic elucidation of gene expression (RNA-Seq), transcription start sites (differential RNA-Seq), transcript 3′ ends (Term-Seq), and post-transcriptional processes (ribosome profiling). The main challenge of transcriptomic studies is to remove ribosomal RNAs (rRNAs), which comprise more than 90% of the total RNA in a cell. Here, we report a low-cost and robust bacterial rRNA depletion method, RiboRid, based on the enzymatic degradation of rRNA by thermostable RNase H. This method implemented experimental considerations to minimize nonspecific degradation of mRNA and is capable of depleting pre-rRNAs that often comprise a large portion of RNA, even after rRNA depletion. We demonstrated the highly efficient removal of rRNA up to a removal efficiency of 99.99% for various transcriptome studies, including RNA-Seq, Term-Seq, and ribosome profiling, with a cost of approximately $10 per sample. This method is expected to be a robust method for large-scale high-throughput bacterial transcriptomic studies.

## Author summary

Removal of ribosomal RNAs, a major constituent (over 90%) of cellular RNA is a critical experimental step for transcriptomic studies that deal with messenger RNAs. In this manuscript, we describe a robust method to subtract ribosomal RNA from various RNA samples. The method is based on the enzymatic degradation of target RNA by short complementary DNA and RNA:DNA duplex specific nuclease. The method comprises carefully designed experimental procedures to minimize experimental bias and unwanted removal of messenger RNAs. We validate the method on various types of transcriptomic studies for seven diverse bacterial species. This method successfully removed ribosomal RNA with over 99% of efficiency and it was comparable to commercial systems even for degraded RNA samples at a fraction of a cost.

This is a *PLOS Genetics* paper.

## Introduction

Genetic information encoded in the genome is transferred to proteins via messenger RNAs (mRNAs). Thus, investigating mRNAs is a central approach to elucidating the fundamentals of cellular functions. Multiple techniques such as quantitative polymerase chain reaction (qPCR), microarray, and RNA sequencing (RNA-Seq) have been developed to quantitatively measure mRNAs inside a cell or their changes in response to a variety of environmental and genetic perturbations [1,2]. Since ribosomal RNAs (rRNAs) comprise more than 90% of the total RNA in a cell, their efficient removal is one of the most important tasks for reliable genome-wide transcriptomics studies [3]. Unlike eukaryotic mRNAs that can be selectively enriched by virtue of their poly A tail structure [4], bacterial and archaeal mRNAs do not, or rarely, possess such features; thus, removal of the predominant rRNA species from total RNA is critical for downstream applications.

Several methods for removing bacterial rRNAs have been developed and commercialized. Subtractive hybridization with nucleic acid probes that are reverse complementary to rRNAs is the most popular method that has been commercialized in multiple systems, such as Ribo-Zero, MICROBExpress, RiboErase, and RiboMinus [5–7]. However, as the listed methods depend on short conserved regions on rRNAs, they tend to be inconsistent in partially degraded RNA samples and species that have divergent rRNA sequences compared to the probe sequence. Exonucleolytic digestion of processed RNA with monophosphate at the 5′ end has also been devised for transcriptomics [8]. However, this method has relatively low efficiency and is limited by the fact that primary transcripts protected by triphosphate from 5′-phosphate-dependent terminator exonuclease (TEX) may not be a precise representation of mRNA levels in a cell, because a considerable amount of mRNA exists as a processed form. Several methods have been proposed for rRNA removal based on duplex-specific nuclease-based digestion, electrophoretic size selection, and sequence-specific blockage of reverse transcription, although they are not as efficient as commercial systems [9–12].

Ribonuclease H (RNase H) is an endoribonuclease that specifically digests RNA strands of RNA:DNA hybrids. The RNase H-based selective digestion of rRNAs has been proposed as a cost-effective alternative method for depleting cellular rRNAs because of its high removal efficiency [3,13]. An RNA-Seq study of formalin-fixed paraffin-embedded (FFPE) archival tissue using RNase H was reported previously [3]. However, this method was not revisited in bacterial samples until the recent temporal discontinuation of the Ribo-Zero. Although an rRNA depletion method was assessed for bacterial RNA-Seq as a low-cost alternative [13], additional optimization is required for efficient rRNA depletion. These optimization methods include the following: (i) optimization of reaction conditions to prevent nonspecific hybridization; (ii) design of the oligonucleotide probes complementary to the rRNAs; (iii) addition of probe specific for premature rRNAs (pre-rRNAs) that contain extended sequences from the operonic structure of rRNA operons; and (iv) application of the method to other transcriptomic studies such as sequencing the 3′ ends of bacterial transcripts (Term-Seq) and ribosome profiling. To this end, we revisited the rRNA depletion method to improve and streamline the experimental procedure by adjusting the reaction temperature, ionic strength, removal of pre-rRNAs, and redesigning probe sequences. The advanced method presented here, called RiboRid, could remove bacterial rRNAs up to 99.99% and prevent nonspecific binding of oligonucleotide probes to other cellular RNA species. Furthermore, RiboRid has rRNA removal efficiency comparable to the most efficient commercial method (Ribo-Zero) when performing technically challenging transcriptomic studies, such as Term-Seq and ribosome profiling.

## Results

### Sequence specific digestion of ribosomal RNAs

**Fig 1:**
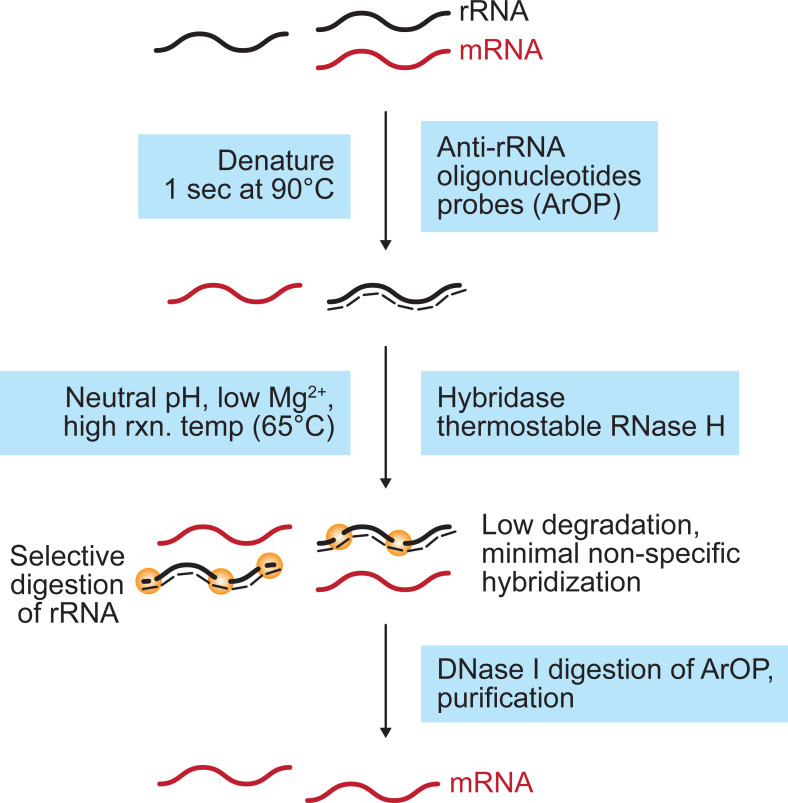
Experimental workflow of RiboRid. Anti-rRNA oligonucleotide probes (ArOPs) are chemically synthesized. Short denaturation time, neutral pH, low Mg^2+^ concentration, and high reaction temperature provides minimal experimental bias.

Selective digestion of rRNAs using RNase H depends on hybridization of the short deoxynucleoside probes specific to rRNAs and digestion of the RNA:DNA hetero-duplex by RNase H. Thus, minimization of the nonspecific hybridization of the probes to other RNA species is critical for efficient and nonbiased transcriptome studies. Thus, the hybridization reaction of the Ribo-Zero method is carried out at a high temperature (68°C) to reduce nonspecific hybridization. To this end, we aimed to optimize the experimental conditions in the RNase H-based rRNA depletion method to prevent unwanted RNA degradation by nonspecific binding (**Fig 1**). First, the RNA sample was denatured at 95°C for only 1 s. Prolonged incubation at a denaturing temperature that may induce spontaneous hydrolysis of RNA was not necessary for all tested samples in this study. Next, we performed RNase H reaction at an elevated temperature (65°C) to prevent unwanted hybridization of anti-rRNA oligonucleotide probes (ArOPs) to mRNA. To support this reaction, Hybridase thermostable RNase H, that has an optimum reaction temperature at 65°C or higher, was used. Synthetic single-stranded deoxyoligonucleotides with melting temperatures higher than 68°C were used as ArOPs to support efficient hybridization at a high reaction temperature (65°C).

We used 32 nt-long single-stranded deoxyoligonucleotides as ArOPs that can bind to rRNA. When the melting temperature of the probe is lower than 68°C, the position of the probe is moved to the rRNA until the melting temperature reaches 68°C. However, when an appropriate probe could not be found or the adjusted probe overlapped with the adjacent probe, the length of the probe was shortened to 28 nt or extended up to 43 nt (see **Methods**). The probes were designed to bind approximately every 40 bp of rRNA and hybridized to rRNA in an RNA sample that had been denatured. Then, the RNA strand of RNA:DNA hetero-duplexes were subjected to RNase H digestion. Compared to the previous approaches, that use mild temperatures (22–45°C) for the digestion reaction [3,13], RNA is relatively prone to spontaneous hydrolysis at the elevated reaction temperature in this study. The nonenzymatic RNA hydrolysis occurs at elevated temperatures due to the presence of magnesium cations (Mg^2+^), which is required for RNase H activity [14]. Thus, the reaction buffer was modified to contain a lower Mg^2+^ concentration, which is 6.4-fold lower than that in previously reported methods [3,13]. With the optimized protocol, we could detect removal of rRNA by electrophoretic analysis (**S1 Fig**), which can be a useful checkpoint before proceeding to downstream study. Subsequently, ArOPs were removed by DNase I digestion followed by column-based purification, to collect remaining mRNAs. This additional enzymatic digestion can be avoided when the 3′ end of ArOP is blocked by C3-spacer or dideoxynucleotide modification, because they are not used as a substrate by ligases and polymerases; thus, they do not interfere with downstream library preparation.

### Streamlining the rRNA removal protocol for RNA-Seq in *Escherichia coli* and closely related species

**Fig 2:**
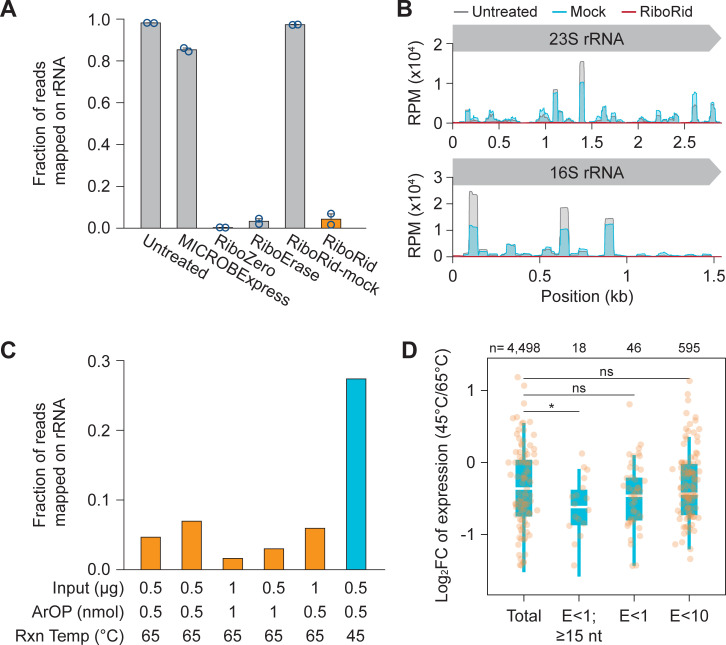
Ribosomal RNA (rRNA) depletion using RiboRid. (A) Efficiency of rRNA removal by commercial kits and the RiboRid method. Error bars indicate the standard deviation of two biological replicates. Circles are individual data points. Untreated: no treatment. Mock: RiboRid treatment with nuclease-free water instead of Hybridase enzyme. (B) RNA sequencing (RNA-Seq) profiles of controls and RiboRid-treated rRNA. Number of reads were counted as reads per million mapped reads (RPM). (C) Efficiency of RiboRid with different experimental conditions (amount of input RNA, amount of anti-rRNA oligonucleotide probes (ArOPs), and temperature). (D) RNA expression levels of genes that have sequence similarity to ArOPs. Genes with sequence similarity longer than 15 nt of consecutive matches with an *E*-value lower than 1 (BLASTN) to ArOPs have significantly low expression levels at low hybridization temperatures (*p = 0.027; Wilcoxon’s rank-sum test). Box limits, whiskers, and center lines indicate 1^st^ and 3^rd^ quartiles, 10^th^ and 90^th^ percentiles, and the median of the distribution, respectively. Dots are individual genes. Number of subjected genes are indicated above the graph.

We first examined the capability of the RiboRid method for removing rRNAs from 500 ng total RNA samples from *Escherichia coli* and compared it with three commercial methods, MICROB*Express*, Ribo-Zero (a legacy system that uses subtractive hybridization), and RiboErase. Without rRNA depletion, rRNA comprised over 97% of the total sequenced reads (**Table 1**; untreated). Commercial kits showed successful removal of rRNA, except for the MICROB*Express* method that showed low removal efficiency as reported elsewhere (**Fig 2A**) [15,16]. The RiboRid method using 108 ArOP sets (**S1 Table**) removed rRNAs from the *E*. *coli* total RNA samples at a level down to 1.63%, which is comparable to commercial methods at a fraction of the cost (**Fig 2A** and **Table 1**). Furthermore, the RiboRid method successfully depleted coverage from the entire 16S and 23S rRNA genes (**Fig 2B**). Technical mock samples that underwent the same RiboRid process with nuclease-free water instead of the Hybridase enzyme, showed virtually no difference to untreated control. In addition, we inspected RNA-Seq profile of highly expressed genes to examine enzymatic reaction introduced biases or RNA degradation as reported earlier [17,18], and did not detect any sign of RNA degradation or bias (**S2 Fig**). In addition, we did not observe non-specific enrichment of short rRNA reads that has been reported in the previous enzyme-based rRNA depletion approach (**S3 Fig**) [17]. Next, we analyzed gene expression by RNA-Seq that are performed with different rRNA removal methods. Except for the untreated and mock samples, all the rRNA-depleted samples and replicates were linearly correlated, even across the different methods (**S4 Fig**). The RNA-Seq dataset prepared from two different biological replicates using the RiboRid method showed high reproducibility, with a correlation of 0.977 (Pearson’s R^2^).

**Table 1:** Fraction of ribosomal RNA in *Escherichia coli* and *Klebsiella oxytoca* RNA-Seq prepared by various rRNA-subtraction methods and experimental conditions. I: input RNA (μg). ArOP: amount of ArOP used (pmol each probes). T: temperature of Hybridase reaction (°C). % indicates fraction of rRNA reads to all reads mapped on genome.

Organism	Method	Note	Mapped reads	Mapped on rRNAs				
				5S rRNA	16S rRNA	23S rRNA	Sub-total	%
*Escherichia coli* MG1655	None	untreated	3,310,864	119	1,744,649	1,507,768	3,252,536	98.24
			3,059,840	61	1,521,945	1,480,521	3,002,527	98.13
	MICROB Express		2,551,754	307	896,617	1,301,681	2,198,605	86.16
			2,184,739	182	719,517	1,127,048	1,846,747	84.53
	Ribo-Zero		6,948,099	4	900	2,979	3,883	0.06
			10,630,331	7	670	2,242	2,919	0.03
	RiboErase		6,289,463	3,297	35,305	75,359	113,961	1.81
			6,703,891	3,224	87,175	197,847	288,246	4.30
	RiboRid	Standard method (I1.0-ArOP5-T65)	5,832,388	25	22,889	62,093	85,007	1.46
			6,107,778	37	129,249	275,698	404,984	6.63
	RiboRid	without Hybridase (mock)	1,949,301	23	832,406	1,062,779	1,895,208	97.23
			1,589,651	18	688,612	859,085	1,547,715	97.36
		I0.5-ArOP5-T65	3,398,659	2,310	106,652	134,399	243,361	7.16
			2,162,423	2,150	46,380	56,302	104,832	4.85
		I1.0-ArOP10-T65	1,239,187	659	8,236	11,761	20,656	1.67
		I0.5-ArOP10-T65	2,304,011	1,367	30,127	39,531	71,025	3.08
		I1.0-ArOP5-T65	1,274,201	764	33,805	43,352	77,921	6.12
		I0.5-ArOP5-T45	2,012,043	584	318,343	238,087	557,014	27.68
*Escherichia coli* MG1655	RiboRid	Standard method	12,984,470	25,927	79,291	66,971	172,189	1.34
			12,019,200	19,354	108,427	144,759	272,540	2.28
		SPRI bead purification, C3 spacer-modified 55 ArOP	23,361,967	47,624	42,410	55,477	145,511	0.63
			21,201,607	44,499	94,442	205,618	344,559	1.64
*Klebsiella oxytoca* KCTC1686	RiboRid	Anti-*E*. *coli* ArOP	3,784,202	3,331	42,380	49,299	95,010	2.51
			2,029,169	2,222	12,972	12,332	27,526	1.36

The number of genes observed by RNA-Seq was also significantly increased by rRNA removal. From the RNA-Seq result of Ribo-Zero, RiboErase, or RiboRid treated samples, expressions of 4,201, 4,166, and 4,122 genes were observed, respectively, while only 2,738 genes were detected in the control samples.

Furthermore, by the nature of the Poisson process of RNA-Seq, gene expression levels measured by multiple replicates showed heteroscedasticity; that is, the variance of gene expression measured between replicates increases as the expression level decreases [19,20]. The variation of biological replicates is a combination of technical and biological variation [19]. Given the experimental setup, the difference in observed variations across different methods were originated from different technical variations, since the libraries were prepared from aliquots of the same biologically replicated samples. Thus, we expected to observe different distribution of variation across expression level if rRNA removal method was unsuccessful or introducing bias. The variation of gene expressions from the samples prepared without rRNA depletion was much higher than that of the rRNA-depleted samples and was not heteroscedastic (**S5 Fig**). In contrast, samples prepared using Ribo-Zero, RiboErase, and RiboRid had identical distribution of experimental variations. Moreover, the average coefficient of variation (standard deviation divided by mean expression of a gene, which implies that the variance of measured expression adjusted by expression level) of the control samples was more than 3.6-fold higher than that of the rRNA-depleted sample, while the three methods had similar values. Considering the number of genes detected and the distribution of experimental variances, rRNA depletion greatly reduced the counting error of RNA-Seq, especially for genes with low abundance.

Next, we tested RNA input up to 1 μg and different ratios of ArOPs to total RNA. There was virtually no difference in the rRNA content of sequenced reads, indicating that the rRNA depletion method works for various amounts of input RNA and ratios of ArOPs to RNA (**Fig 2C**). In addition, correlations between these technically replicated samples were higher than 0.989 (**S6A Fig**). This shows that the method is sufficiently robust to accommodate practical variations in biological experiments.

To examine the possible nonspecific binding of ArOPs to mRNA, we repeated RNA-Seq from the same RNA sample using the RiboRid method at 45°C (**Fig 2C**). Although the RiboRid method was able to remove rRNA, the fraction of reads from rRNA increased to 27.4% because of the suboptimal reaction temperature of Hybridase. In addition, correlations of the samples with other technical replicates were relatively low (Pearson’s R^2^ = 0.744 ± 0.019) (**S6B Fig**). More importantly, we predicted 46 nonspecific bindings of ArOPs to mRNA (*E*-value < 1 using the Basic Local Alignment Search Tool (BLAST) (**S2 Table**). Eighteen of the probes had consecutive matches longer than 15 bp. Expression of the 18 genes measured by the RNA-Seq library prepared from the rRNA-subtracted RNA sample using the RiboRid method at 45°C was significantly underestimated when compared to the normal RiboRid-treated sample (*p =* 0.027; Wilcoxon’s rank-sum test) (**Fig 2D**). In contrast, we did not observe any significant difference in gene expression between untreated, mock, and RiboRid treated samples (**S7 Fig**). This illustrates the importance of a high reaction temperature in preventing nonspecific binding of probes to mRNA and demonstrates that high reaction temperature of RiboRid was able to avoid experimental bias.

**Fig 3:**
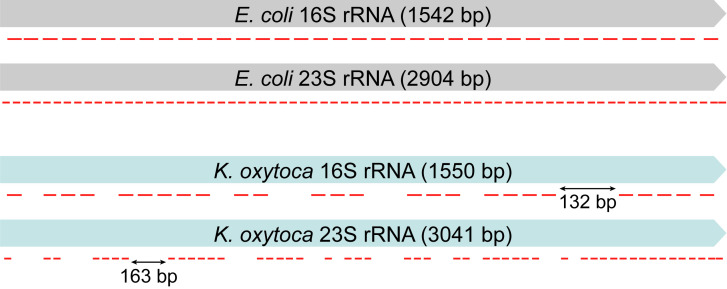
Alignment of *Escherichia coli* anti-rRNA oligonucleotide probes (ArOPs) to *E*. *coli* or *Klebsiella oxytoca* ribosomal RNAs (rRNAs). Red bars indicate 32-nt ArOPs.

Next, we applied the method and the same ArOPs to RNA-Seq of *K*. *oxytoca* KCTC1686, a bacterial species closely related *to E*. *coli*. Owing to the sequence similarity between the two bacteria, 76 out of 108 anti-*E*. *coli* ArOPs matched the rRNAs of *K*. *oxytoca* (**Fig 3**). Although gaps in *K*. *oxytoca* rRNAs, where no ArOP hybridizes, span a maximum length of 163 nt, 76 ArOPs were dense enough to remove rRNAs to a level less than 3% of mappable sequence reads in *K*. *oxytoca* RNA-Seq, with a correlation of 0.998 (Pearson’s R^2^) between biological replicates (**Table 1**). rRNA removal of *K*. *oxytoca* using anti-*E*. *coli* ArOPs indicates that the number of ArOPs may be reduced to a much lower density. Thus, we designed a new probe set in which each probe was spaced at a longer distance. The new probe set consisted of 55 probes that could hybridize approximately every 80 nt on the rRNAs (**S3 Table**). We also adjusted the protocol further to reduce the cost and time of the RiboRid method. First, ArOPs were synthesized with a C3 spacer at the 3′ end, which is not utilized as a substrate for ligases and polymerase in downstream library construction. Originally, oligonucleotide probes remained after the RiboRid reaction needed to be removed by DNase digestion, since they can interfere with the downstream PCR amplification step of sequencing library construction. However, C3 spacer-modified oligonucleotides cannot be used as primers for DNA polymerase; thus, DNase I digestion of probes at the end of the RiboRid method can be avoided. Alternatively, the 3′ end of pre-existing probes can be blocked by attaching dideoxynucleotide analogs using terminal deoxynucleotidyl transferases instead of C3 spacer modification. Then, the column-based clean-up step was replaced by SPRI paramagnetic bead purification. Together with these modifications, the cost and processing time of the RiboRid method can be reduced considerably (**S1** and **S2 Protocol**) without increasing the rRNA fraction or experimental bias (**Table 1** and **S6C Fig**).

### Depletion of rRNA from the RNA samples from various bacterial species

**Fig 4:**
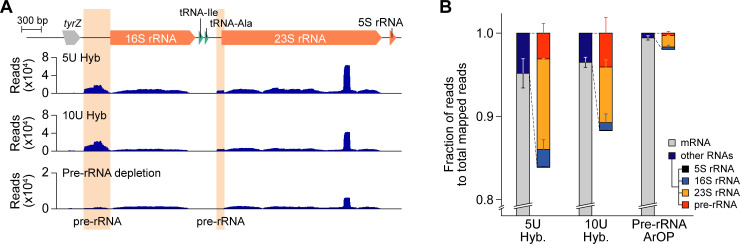
Pre-ribosomal RNA (rRNA) content in RNA sequencing (RNA-Seq) experiments of *Pseudomonas aeruginosa*. (A) RNA-Seq profile of rRNA operon in *P*. *aeruginosa*. Shaded area indicates premature rRNA fragment. 5U Hyb: RNA-Seq prepared by treating standard RiboRid reaction (standard amount of Hybridase; 5 units). 10U Hyb: sample treated with twice more Hybridase than standard method (10 units). Pre-rRNA depletion: RNA-Seq profile of sample prepared by RiboRid reaction comprising 10 additional ArOP targeting pre-rRNA species. (B) Fraction of reads mapped on mRNA and other rRNA-related RNAs. Error bars indicate the standard deviation of two biological replicates.

To examine the versatility of the RiboRid method for diverse bacterial species, we applied RiboRid to two Gram-negative bacteria, *Pseudomonas aeruginosa* and *Bacteroides thetaiotaomicron*, and two Gram-positive bacteria, *Eubacterium limosum* and *Staphylococcus aureus*, with ArOPs specific for each bacterium (**Table 2**). *P*. *aeruginosa* is a Gram-negative opportunistic pathogen that is a common cause of pneumonia and other infections in various parts of the body [21]. rRNAs were successfully removed from the laboratory derivative PAO1 isolate [22] using the standard RiboRid method to a level where rRNA comprised an average of 4% of mappable reads in RNA-Seq. However, we encountered an interesting profile of rRNA operons (**Fig 4A**). There were large amounts of RNA fragments in the intergenic region of the rRNA operons. Considering rRNA maturation and processing in bacteria, the fragments were predicted to be the pre-rRNAs that were not depleted due to the absence of complementary ArOPs. The pre-rRNAs comprised an average of 0.84% of the mapped reads in RNA-Seq. Although the amount of pre-rRNA reads was negligible when compared to mRNA reads, we tested two different approaches to further remove pre-rRNA species. First, a two-fold increase in Hybridase in the reaction did not affect the overall amount of non-mRNA or pre-rRNA reads (**Fig 4A and 4B**). In fact, supplementation of a few anti-pre-rRNA oligos (**S4 Table**) in the Hybridase reaction reduced the amount of pre-rRNA fragments from the RNA sample to 0.06% (**Fig 4B**).

**Table 2:** Fraction of rRNA in RNA-Seq of multiple bacterial species, in which rRNA were removed by the RiboRid method. % indicates the fraction of rRNA reads of all mapped reads of the genome

Organism	Note	Mapped reads	Mapped on rRNAs				
			5S rRNA	16S rRNA	23S rRNA	Sub-total	%
*Pseudomonas aeruginosa*	5 U Hybridase	26,174,448	4,500	312,841	1,164,705	1,482,046	5.66
		23,088,094	3,399	56,816	479,347	539,562	2.34
	10 U Hybridase	28,748,624	3,954	167,246	737,542	908,742	3.16
		29,991,165	3,557	13,083	452,044	468,684	1.56
	with pre-rRNA probes	19,868,429	1,106	4,442	46,126	51,674	0.26
		21,443,360	889	41,453	125,739	168,081	0.78
*Staphylococcus aureus*	Medium (CAMHB)	16,321,596	9	19418	35296	54,723	0.34
		16,852,709	112	105020	185302	290,434	1.72
	Medium (RPMI)	14,734,909	2	694	1026	1,722	0.01
		15,889,282	2	1592	3470	5,064	0.03
*Eubacterium limosum*	Standard method	8,079,397	563	63,851	117,606	182,020	2.25
		9,107,085	479	125,218	229,290	354,987	3.90
*Bacteroides thetaiotaomicron*	ArOP set 1	2,118,423	0	275,845	406,929	682,774	32.23
		2,528,261	0	221,023	339,672	560,695	22.18
	ArOP set 2	7,480,373	0	746,822	589,762	1,336,584	17.87
		9,225,721	0	934,332	705,684	1,640,016	17.78
	ArOP set 2 with rxn. Temp. 58°C	1,952,549 1,136,665	0 0	75,339 134,350	59,576 124,706	134,915 259,056	6.91 22.79

We further applied the RiboRid method to two Gram-positive bacteria: *S*. *aureus*, a common pathogen that is one of the biggest threats to the health care system due to the emergence of strains with antibiotic resistance [23,24]; and *E*. *limosum*, a model strain of CO_2_-fixing acetogenic bacteria [25]. rRNAs from both strains were efficiently removed by the RiboRid method (**Table 2**). The fraction of *S*. *aureus* rRNAs in RNA-Seq was lower than 1.72% regardless of the culture medium, with Pearson’s correlation (R^2^) between biological replicates higher than 0.955 (**S8 Fig**). In particular, rRNAs comprised only 0.02% of the total mapped reads when RNA was prepared from the cells grown in RPMI medium. The RiboRid method was also capable of reducing rRNAs from *E*. *limosum* down to 2.25% with high reproducibility (R^2^ = 0.949) between biological replicates (**Table 2**).

*B*. *thetaiotaomicron* is one of the major constituents of the human gut commensal microbiome [26,27]. Unlike in other bacterial species, the RiboRid method left relatively high amount of rRNA from RNA sample of *B*. *thetaiotaomicron*. In detail, rRNA from the RiboRid-treated sample comprised 27.20 ± 5.03% (**Table 2**), although the RNA expression measured from RiboRid-treated sample was highly correlated with that of Ribo-Zero-treated samples with a Pearson’s correlation (R^2^) of 0.915 ± 0.01 (**S8 Fig**). To improve the efficiency of rRNA removal, we designed a new ArOP set (ArOP Set 2) with different DNA sequences that have higher melting temperatures, so that they can hybridize to rRNA stronger at the elevated reaction temperature of RiboRid. Although the fraction of rRNA to the total mapped reads was reduced to 17.82 ± 0.05%, its correlation with the Ribo-Zero- or standard ArOP (ArOP Set 1)-treated sample was decreased to 0.741 (R^2^) (**S8 Fig**). Lowering the reaction temperature to 58°C, which is at least 10°C lower than the annealing temperature, did not improve the rRNA removal efficiency (**Table 2**). A recent report also indicated that both Ribo-Zero and RNase H-based removal of rRNAs *Bacteroidetes dorei*, were relatively inefficient, possibly due to its high genomic AT-content [13]. As illustrated by the duplex-specific nuclease-based rRNA removal method [16], further experimental and probe optimization may be required for organisms with high AT content, such as *Bacteroidetes*; however, the RiboRid method was able to remove rRNAs to a level less than 20% of total RNA.

### Application of the RiboRid method to Term-Seq

**Fig 5:**
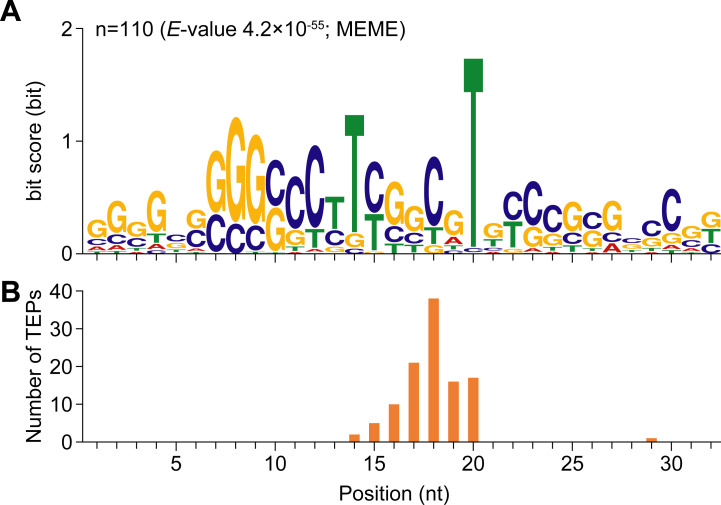
A conserved rho-independent terminator identified from the upstream sequence of the transcript 3′ end positions (TEPs). (A) The 32-nt terminator motif conserved upstream of 110 TEPs comprises a GC-rich stem followed by a U-rich tract. (B) Position of TEPs on the motif.

To further validate the RiboRid method for other challenging transcriptomic analysis methods, we conducted Term-Seq [28]. The Term-Seq technique captures the 3′ ends of transcripts in a cell at the nucleotide level [28]. Thus, it is sensitive to RNA degradation, which generates false signals. We successfully detected 1,308 transcript 3′ ends across the transcriptome of the model actinomycetes, *S*. *coelicolor*, which is a Gram-positive soil bacterium well-known for producing various secondary metabolites [29]. Reads mapped on rRNAs comprised only 0.36% of the reads mapped to the genome (**Table 3**). To address possible non-specific random RNA degradation during the RiboRid treatment that may decrease resolution of single nucleotide-sensitive study such as Term-Seq, we inspected profile of Term-Seq (**S9 Fig**). For example, transcript 3′ end detected from two biological replicates coincides with each other in nucleotide-level (**S9A** and **S9B Fig**). Furthermore, the Term-Seq signal from two replicates were highly correlated (Pearson’s R^2^ of 0.9998) across the entire genome (**S9C Fig**). Thus, we concluded that RiboRid did not introduce any noise for the transcriptomic study. With successful depletion of rRNAs from total RNA of *S*. *coelicolor* with high GC content (72.4%), we were able to identify a conserved rho-independent transcriptional terminator motif at the 110 transcript 3′ ends, which resembles the rho-independent transcriptional terminators of the closely related actinomycetes, *S*. *lividans* (**Fig 5A**) [30]. In addition, the location of transcription termination occurred on the T-rich tract of the conserved motif, which was consistent with the previous report (**Fig 5B**) [30]. This demonstrates that the RiboRid method can be applied to transcriptome sequencing other than RNA-Seq.

**Table 3:** Fraction of rRNA in Term-Seq and ribosome profiling prepared with RiboRid. % indicates the fraction of rRNA reads of all mapped reads of the genome.

Organism and experiment	Note	Mapped reads	Mapped on rRNAs				
			5S rRNA	16S rRNA	23S rRNA	Sub-total	%
*Streptomyces coelicolor* Term-Seq	Standard method	6,435,085	80	9,911	18,072	28,063	0.44
		3,229,425	27	2,724	6,654	9,405	0.29
*Escherichia coli* ribosome profiling	Ribo-Zero	72,406,373	8,511	28,603,979	27,456,111	56,068,601	77.44
		100,774,910	10,569	31,910,262	40,093,315	72,014,146	71.46
		48,475,872	8,405	11,149,390	23,766,472	34,924,267	72.04
	Standard method	2,056,321	75,394	1,454,309	406,072	1,935,775	94.14
		3,207,956	52,944	2,725,659	335,259	3,113,862	97.07
	ArOP + additional set 1	1,231,074	3,635	182,472	689,857	875,964	71.15
		2,206,290	6,101	260,871	1,422,499	1,689,471	76.58
	ArOP + additional set 1 & 2	8,941,114	45,608	3,214,429	3,159,559	6,419,596	71.80
		3,185,119	13,277	1,109,650	1,019,660	2,142,587	67.27
		2,214,451	10,464	603,139	857,434	1,471,037	66.43
		2,135,544	16,454	678,992	908,681	1,604,127	75.12

### Application of the RiboRid method to ribosome profiling

**Fig 6:**
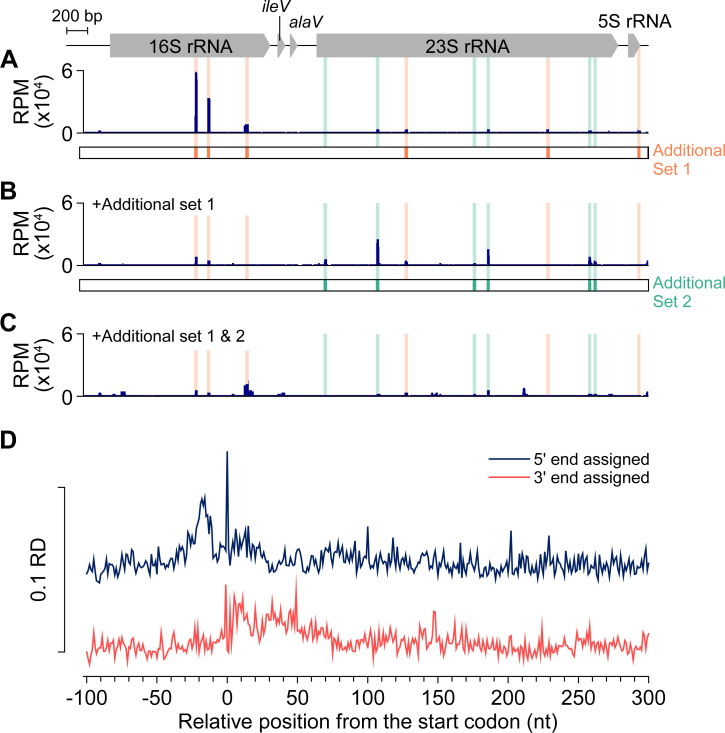
Ribosome profile on one of the ribosomal RNA (rRNA) operons in *Escherichia coli*. (A) Enriched rRNA fragments of ribosome profile prepared from an RNA sample treated with standard RiboRid. Orange bars show additional oligonucleotide probes (set 1) targeting the fragments. (B) Ribosome profile of RNA sample prepared using RiboRid with additional probe set 1. Green bars show newly enriched regions and additional probe set 2. (C) Two additional probe sets specific for the enriched ribosome-protected fragments (shaded area) could effectively remove the fragments. RPM: number of reads per million mapped reads. (D) Meta-analysis of ribosome profile from RiboRid-treated sample aligned at the start codon by different read assignment method. Either 5′ or 3′ ends of sequencing reads were used to determine boundary of ribosome. Ribosome density (RD) is average ribosome profile of coding sequences normalized by dividing ribosome profile of each positions with the maximum peak height in 400 nt window.

Ribosome profiling is a transcriptome sequencing technique that surveys mRNAs that are actively translated by capturing ribosome-protected fragments (RPFs) [31]. It is one of the most challenging transcriptome analysis techniques because it generates highly fragmented short rRNA fragments during enzymatic digestion of RNA that are not protected by ribosomes. Depletion of highly fragmented short rRNA fragments is a major technical challenge in bacterial ribosome profiling. We performed ribosome profiling of *E*. *coli* using the RiboRid method, which was modified by adopting a previously developed streamlined protocol (see **Methods**) [32]. Initially, the method failed to remove rRNA fragments from the RPFs, such that 95.6% of sequencing reads were mapped to rRNA (**Table 3**). Close inspection of the sequencing profile indicated that specific regions of rRNA were enriched (**Fig 6A**). Thus, we designed and supplemented six additional probes (additional set 1) targeting the enriched regions (**S5 Table**). The specific RNA fragments were clearly depleted after RiboRid treatment with the additional probe set 1 (**Fig 6B**). However, another rRNA fragment remained and became more noticeable relative to the fragments targeted by probe set 1. We constructed six additional probes (additional set 2) and RiboRid with the two additional probe sets reduced the fraction of reads mapped on rRNA down to the level shown by the Ribo-Zero-treated sample, which was approximately 70% (**Fig 6C** and **Table 3**). Meta-analysis of the ribosome profile on coding sequences showed a nucleotide-resolution profile of ribosome footprint and pausing on the start codon (**Fig 6D**), which is comparable to Ribo-Zero treated profile (**S10 Fig**), illustrating the capability of RiboRid in ribosome profiling [33].

Taken together, RiboRid effectively removed the fraction of rRNA even from highly fragmented polysomal RNA samples, indicating that it can be used for complicated transcriptomic studies other than RNA-Seq. The single base-pair resolution required for probing 3′ end information and the precise polysomal location on the RNA were not compromised by the method developed in this study.

## Discussion

Based on the transcriptomic sequencing results presented in this study, we demonstrated a strategy for effectively depleting rRNA from bacterial RNA samples. The RiboRid method is highly advanced and effective method, capable of removing rRNA from highly fragmented RNA samples without losing precise biological information of the mRNA. Owing to thorough experimental and protocol design considerations regarding hybridization temperature and probe design, the prevalent rRNA species in bacterial RNA samples could be efficiently removed without introducing any experimental bias or causing nonspecific mRNA removal. Furthermore, a short incubation time at denaturing temperature, low concentration of divalent magnesium cations, and mild pH prevented RNA degradation observed in the previous enzymatic approaches [17,18]. More importantly, the high hybridization and reaction temperature prevented nonspecific binding of probes to mRNA that may occur in the previous approaches using low hybridization temperature [13]. Optimization of the probe number could streamline the protocol such that the cost is dramatically reduced ($10 per reaction) when compared to the commercially available method (Ribo-Zero; ~$80 per reaction), with comparable or better rRNA removal efficiency. Although this method is based on enzymatic RNA digestion, the total experiment time is only 1 h with a hands-on time of 30 min; this time is comparable to the hybridization-magnetic subtraction-based method.

RiboRid is highly efficient and could remove rRNA with an efficiency of up to 99.99% in the *S*. *aureus* sample. However, in the case of *B*. *thetaiotaomicron*, rRNA comprised 17% of all mappable rRNA reads. This is possibly because of the high AT content of the organism, in which hybridization of the probe at high temperature may be inefficient. However, this can be easily compensated for by using a slightly higher sequencing depth, as the sequencing cost is much lower than the difference between the costs of the RiboRid and commercial methods.

To facilitate the use of this method, a step-by-step protocol is available in the S1 Protocol. The protocol contains all the consumables, materials, and equipment required to perform the RiboRid reaction. In addition, an in-house Python script is freely available through a public repository (https://github.com/SBRG/RiboRid_Design); this script can design a probe set for any custom genome or rRNA sequence. In addition, we designed and deposited probe sets for representative bacterial species available in the RefSeq database (n = 5,467). This method provides a cost-effective, rapid, and powerful alternative means to deplete rRNA that outperforms previously developed and reported methods. In particular, this method is valuable for routine large-scale transcriptome studies and reduces the burden of high-cost commercial kits.

## Methods

### Bacterial strains and cultivation

Four Gram-negative strains (*Escherichia coli* MG1655, *Klebsiella oxytoca* KCTC 1686, *Pseudomonas aeruginosa* PAO1, and *Bacteroides thetaiotaomicron* VPI-5482) and three Gram-positive strains (*Staphylococcus aureus* pulsotype USA300 strain TCH1516, *Eubacterium limosum* ATCC 8486, and *Streptomyces coelicolor* M145) were used in this study. *E*. *coli* was grown in Luria-Bertani (LB) broth at 37°C. *K*. *oxytoca* KCTC 1686 was grown at 37°C in T1 semi-defined xylose medium (5 g/L yeast extract, 6.6 g/L (NH_4_)_2_SO_4_, 8.7 g/L K_2_HPO_4_, 6.8 g/L KH_2_PO_4_, 0.25 g/L MgSO_4_⋅7H_2_O, 0.05 g/L FeSO_4_⋅7H_2_O, 0.001 g/L ZnSO_4_⋅7H_2_O, 0.001 g/L MnSO_4_⋅H_2_O, 0.001 g/L CaCl_2_⋅2H_2_O, and 20 g/L xylose). *P*. *aeruginosa* was grown at 37°C in M9 medium (47.75 mM Na_2_HPO_4_, 22.04 mM KH_2_PO_4_, 8.56 mM NaCl, 18.70 mM NH_4_Cl, 2 mM MgSO_4_, and 0.1 mM CaCl_2_) supplemented with 2 g/L succinate. *S*. *aureus* was cultured in cation-adjusted Mueller Hinton Broth (CA-MHB) or Roswell Park Memorial Institute (RPMI)-1640 medium supplemented with 10% LB medium (R10LB). The CA-MHB contained 2 g/L beef infusion solids, 1.5 g/L starch, 17.5 g/L casein hydrolysate, 25 mg/L calcium, and 12.5 mg/L magnesium. *E*. *limosum* was cultivated at 37°C in DSM135 medium comprising 1 g/L NH_4_Cl, 2 g/L yeast extract, 10 g/L NaHCO_3_, 0.1 g/L MgSO_4_⋅7H_2_O, 0.3 g/L cysteine-hydrochloride, 10 mL vitamin solution (4 mg/L biotin, 4 mg/L folic acid, 20 mg/L pyridoxine-HCl, 10 mg/L thiamine-HCl, 10 mg/L riboflavin, 10 mg/L nicotinic acid, 10 mg/L pantothenate, 0.2 mg/L vitamin B_12_, 10 mg/L *p*-aminobenzoic acid, and 10 mg/L lipoic acid), 4.6 mM KH_2_PO_4_, 5.4 mM K_2_HPO_4_, 4 μM resazurin, and 20 mL trace element solution (1.0 g/L nitrilotriacetic acid, 3.0 g/L MgSO_4_⋅7H_2_O, 0.5 g/L MnSO_4_⋅H_2_O, 1.0 g/L NaCl, 0.1 g/L FeSO_4_⋅7H_2_O, 180 mg/L CoSO_4_⋅7H_2_O, 0.1 g/L CaCl_2_⋅2H_2_O, 180 mg/L ZnSO_4_⋅7H_2_O, 10 mg/L CuSO_4_⋅5H_2_O, 20 mg/L KAl(SO_4_)_2_⋅12H_2_O, 10 mg/L H_3_BO_3_, 10 mg/L Na_2_MO_4_⋅2H_2_O, 30 mg/L NiCl_2_⋅6H_2_O, 0.3 mg/L Na_2_SeO_3_⋅5 H_2_O, 0.4 mg/L Na_2_WO_4_⋅2H_2_O), supplemented with 5 g/L glucose. *E*. *limosum* was cultured anaerobically in 150-mL serum bottle containing 100 mL culture medium purged with N_2_ gas at a pressure of 200 kPa. *B*. *thetaiotaomicron* was cultured in brain-heart infusion-supplemented broth (BHIS). One liter of BHIS broth contains 37 g Brain-Heart Infusion (BD Difco, Franklin Lakes, NJ, USA), 5 g yeast extract, 0.5 g/L cysteine hydrochloride monohydrate, 0.2 mM L-histidine, 1.9 μM hemin, and 1 μg/mL menadione and was adjusted to pH 8. Cells were cultured in a 150-mL serum bottle containing 100 mL culture media purged with N_2_/CO_2_ (90:10) gas at a pressure of 80 kPa at 37°C with agitation. *S*. *coelicolor* was cultured in 50 mL R5 medium at 30°C with 8 g glass beads (diameter of 3 mm) contained in a 250-mL baffled Erlenmeyer flask. One liter of the R5 medium is composed of 5.73 g N-Tris(hydroxymethyl)methyl-2-aminoethanesulfonic acid (TES; pH 7.2), 103 g sucrose, 10 g glucose, 5 g yeast extract, 10.12 g MgCl_2_∙6H_2_O, 0.25 g K_2_SO_4_, 0.1 g casamino acids, 0.08 mg ZnCl_2_, 0.4 mg FeCl_3_∙6H_2_O, 0.02 mg CuCl_2_∙2H_2_O, 0.02 mg MnCl_2_∙4H_2_O, 0.02 mg Na_2_B_4_O_7_∙10H_2_O, and 0.02 mg (NH_4_)_6_Mo_7_O_24_∙4H_2_O.

### RNA extraction

Total RNA of *E*. *coli*, *K*. *oxytoca*, and *B*. *thetaiotaomicron* were extracted using the RNASnap method [34] with slight modifications. Briefly, cell pellets collected from 5 mL mid-exponentially grown culture (OD_600nm_ = 0.4) was resuspended in 100 μL RNASnap solution (18 mM EDTA, 0.025% SDS, 1% β-mercaptoethanol, and 95% formamide) and incubated at 95°C for 7 min. The resuspension was centrifuged at 16,000 × *g* for 5 min and RNA with a size larger than 200 nt in the clear supernatant was purified using RNA Clean & Concentrator-5 Kit (Zymo, Irvine, CA, USA) according to the manufacturer’s instructions. Total RNA of *E*. *limosum* was extracted from 100 mL mid-exponential growth culture (OD_600nm_ = 1.5). The collected cells were resuspended in 500 μL lysis buffer (20 mM Tris-HCl [pH 7.4], 140 mM NaCl, 5 mM MgCl_2_, and 1% Triton X-100) and ground using a mortar and pestle after flash freezing with liquid nitrogen. RNA was isolated from the supernatant of the ground sample using TRIzol Reagent (Thermo, Waltham, MA, USA) followed by an RNA Clean & Concentrator-5 Kit according to the manufacturer’s instructions. Total RNA of *P*. *aeruginosa* and *S*. *aureus* was extracted using Quick-RNA Fungal/Bacterial Microprep Kit (Zymo) from 3 mL culture sampled at an OD_600nm_ of 0.4. Briefly, the cell pellet was resuspended in 800 μL RNA lysis buffer. The resuspension was then transferred into a ZR BashingBead Lysis Tube (0.1 and 0.5 mm) and homogenized in a bead beater. RNA in the cleared lysate (400 μL) was purified by column purification. Total RNA of *S*. *coelicolor* was extracted using the hot phenol method from 50 mL culture samples at the early exponential, transition, late exponential, and stationary growth phases (OD_600nm_ = 0.6, 2, 3.5, and 5, respectively). First, the collected cells were resuspended in Solution 1 (25 mM Tris-HCl [pH 8.0], 10 mM EDTA, 50 mM glucose, and 2 mg/mL lysozyme) and incubated for 10 min at 30°C. The supernatant of the mixture was removed by centrifugation, and the cell pellet was resuspended in Solution 2 (50 mM sodium acetate [pH 5.2], 10 mM EDTA, and 1% sodium dodecyl sulfate). The suspension was mixed with an equal volume of phenol:chloroform (5:1) solution and incubated for 5 min at 65°C. RNA was isolated by isopropanol precipitation from the aqueous phase and separated by centrifugation.

### Anti-rRNA oligonucleotide probes (ArOPs) design

The probes used were 32 nt-long deoxynucleotides reverse complementary to the rRNA with melting temperatures at least 3°C higher than the rRNA digestion reaction temperature (68°C). The anti-rRNA oligonucleotide probes were designed using an in-house Python program (https://github.com/SBRG/RiboRid_Design). The program can be either imported into other pipelines or executed as a standalone program from the command line. As a basic input, the script takes in GenBank files (with annotated rRNA) or a FASTA file containing the rRNA sequences for the target organism. Given the rRNA sequences in either format, the script starts by building a consensus sequences for rRNAs. These consensus sequences were then used to build a DNA probe library. To build the library for each rRNA type, we started from the 50^th^ sequence position of the rDNA. The first probe was defined as the DNA sequence that started from this position and was the length of the user-defined probe. With a default probe length of 32, the probe starts at position 50 and ends at position 81. If the probe has a melting temperature above the defined melting threshold (68°C used in this study), then the script looks for the next probe downstream with the user-defined maximum gap (default of 50 nt) between the probes. Typically, 12–25 probes per kb of rRNA are required for efficient removal of rRNA (one probe per 40–80 nt). The melting temperatures of the probes were calculated using the OligoAnalyzer Tool (IDT, Coralville, IA, USA). If the melting temperature of the probe is below the threshold, then the script will look for another probe up to the user-defined maximum search space (default of 10 bp) upstream of the starting search position and record the probe with the highest melting temperature. Note that the probe with the highest melting temperature was recorded even if the melting temperature of the probe was below the threshold. Following these steps, the script generates probes in a stepwise manner until all the rRNAs are covered. Once the design process is finished, the designed probe sequences are recorded in a FASTA file, and the metadata associated with each probe (containing information such as its melting temperature, start position, end position, etc.) is recorded in a.csv text file. Using the script, the melting temperatures of 32 nt-long probes were higher than 68°C in 98.3% of the design attempts. In a small number of cases (1.7%), the melting temperature criteria were met by manually adjusting the lengths of probes from 28 to 43 nt (especially in the AT-rich pre-rRNA region).

### RiboRid

Before performing rRNA depletion, RNA content was measured using a Qubit RNA HS Assay Kit (Thermo) with a Qubit 2.0 fluorometer (Thermo). RNA extract was subjected to DNase I treatment for removal of DNA contamination at 37°C for 10 min in 15 μL reaction mixture containing 0.5–1 μg total RNA, 1.5 μL 10× DNase I Buffer, and 2 U RNase-free DNase I. Then, DNase I was inactivated at 75°C for 10 min after addition of 15 μL Hybridase Complement Buffer comprising 90 mM Tris-HCl (pH 7.5) and 200 mM KCl. The ArOP mixture (1 μL) containing 5 pmol of each probe and 100 mM MgCl_2_ was added to the DNase I-treated RNA sample. To hybridize the ArOP to RNA, the temperature of the RNA-ArOP mixture was heated to 90°C for 1 s and cooled to 65°C on a thermocycler. When the temperature of the mixture reached 65°C, 10 U Hybridase Thermostable RNase H (Lucigen, Middleton, WI, USA) prewarmed at room temperature was added. The reaction was carried out at 65°C for 20 min and 90°C for 1 s to rehybridize the ArOP to the remaining rRNA, and then at 65°C for an additional 10 min. Then, RNA larger than 200 nt was extracted using RNA Clean & Concentrator Kit. A nucleic acid-binding column was saved for later use. The remaining ArOPs were removed by DNase I treatment in a reaction composed of 10 U RNase-free DNase I and 5 μL 10× DNase I buffer in a total reaction volume of 50 μL. The DNase I reaction was carried out by consecutive 5 min incubations at 25, 30, 35, 40, and 45°C. The reaction was again purified with the RNA Clean & Concentrator Kit using a nucleic acid binding column saved from the previous clean-up. A step-by-step protocol is provided in **S1** and **S2 Protocol**. The column-based RNA purification step can be replaced by a solid-phase reversible immobilization (SPRI) bead-based purification method. In this study, RNA was purified using 1.8 volume of CleanNGS DNA & RNA SPRI Bead (Bulldog Bio, Portsmouth, NH, USA) according to the manufacturer’s instructions. When using a 3′-C3 carbon spacer-modified ArOP that does not participate in downstream reactions (reverse transcription, adaptor ligation, and PCR), the DNase I treatment step for removing residual ArOPs after the hybridase reaction was not performed.

### RNA-Seq

RNA-Seq libraries were constructed from approximately 100 ng rRNA-depleted RNA using a TruSeq Stranded mRNA LT Sample Prep Kit (Illumina, San Diego, CA, USA) or KAPA RNA HyperPrep Kit (Roche, Basel, Switzerland) according to the manufacturer’s instructions. Constructed sequencing libraries were quantified using a Qubit dsDNA HS Assay Kit with a Qubit 2.0 fluorometer and a TapeStation 2200 (Agilent, Santa Clara, CA, USA) equipped with a High Sensitivity D1000 Screen Tape (Agilent). The library was sequenced using an Illumina platform. Information on NGS (run recipe and instrument) is summarized in **S6 Table**.

### Term-Seq

Total RNA extract containing 5 μg total RNA was treated with DNase I at 37°C for 15 min in a 50 μL reaction comprising 2 U RNase-free DNase I and 5 μL 10× DNase I buffer. The Term-Seq library was constructed as previously described [29]. Briefly, 5′-DNA adaptor was ligated to 1 μg of the pooled RNA in 25 μL reaction mixture containing 150 pmol amino-blocked 3′-DNA adaptor (5′-p-NNAGATCGGAAGAGCGTCGTGT-AmMO), 25 U T4 RNA Ligase 1 (NEB, Ipswich, MA, USA), 2.5 μL 10× T4 RNA Ligase 1 Buffer, 2.5 μL 10 mM ATP, 2 μL dimethyl sulfoxide, and 9.5 μL polyethylene glycol 8000. The reaction mixture was incubated for 2.5 h at 23°C followed by RNA purification using 2.2× volumes of AMPure XP Beads (Agencourt, Beverly, MA, USA). The adaptor-ligated RNA was then subjected to the column-based RiboRid method as described above. Then, rRNA-depleted RNA was fragmented by incubating at 72°C for 90 s with 1 μL RNA Fragmentation Reagent in a total reaction volume of 10 μL, followed by RNA purification using 2.2× volumes of AMPure XP Beads. Then, cDNA was synthesized from rRNA-depleted RNA using 200 U SuperScript III Reverse Transcriptase and 10 pmol amino-blocked reverse transcription primer (5-TCTACACTCTTTCCCTACACGACGCTCTTC) in a total reaction volume of 30 μL. The synthesized cDNA was purified with 1.5× volumes of AMPure XP Beads. Then, cDNA 3-adaptor (5-p-NNAGATCGGAAGAGCACACGTCTGAACTCCAGTCAC-AmMO) was ligated into the 3-DNA adaptor ligation mixture for 8 h at 23°C. The ligation product was purified using 1.8× volumes of AMPure XP Beads and amplified using Phusion High-Fidelity DNA Polymerase (Thermo). The library was sequenced using an Illumina platform. Information on NGS (run recipe and instrument) is summarized in **S6 Table**.

### Ribosome profiling using Ribo-Zero

Ribosome profiling was conducted using a method described in a previous report [32]. Briefly, 50 mL *E*. *coli* culture was collected after 5 min of treatment with chloramphenicol (34 mg/mL) at an exponential growth phase. Cells were flash frozen with 0.5 mL lysis buffer (1% Triton X-100, 34 μg/mL chloramphenicol, 133 mM NaCl, 4.75 mM MgCl_2_, and 19 mM Tris-HCl, pH 7.5) and lysed using a mortar and pestle. Then, the supernatant containing 100 μg RNA was treated with 2,000 gel units of micrococcal nuclease (MNase; NEB). Polysomes were recovered from MNase-digested samples using Illustra MicroSpin S-400 HR columns (GE Healthcare, Chicago, IL, USA) followed by phenol:chloroform:isoamyl alcohol extraction. rRNA was removed from 1 μg polysome-protected RNA using a Ribo-Zero rRNA Removal Kit according to the manufacturer’s instructions. The rRNA-subtracted RNA samples were phosphorylated by treating 10 U T4 polynucleotide kinase (NEB) at 37°C for 1 h and purified with RNeasy MinElute columns (Qiagen, Hilden, Germany). Sequencing libraries were prepared from phosphorylated RNA samples using the NEBNext Small RNA Library Prep Set for Illumina (NEB) according to the manufacturer’s instructions. The library was sequenced using an Illumina platform. Information on NGS (run recipe and instrument) is summarized in **S6 Table**.

### Ribosome profiling using RiboRid

Polysomal RNA was prepared as described previously. The polysomal RNA samples were subjected to phosphorylation without rRNA removal by treating with 10 U T4 polynucleotide kinase at 37°C for 1 h and purified with RNeasy MinElute columns. 5′ sequencing adaptors were attached to phosphorylated RNA samples using the NEBNext Small RNA Library Prep Set for Illumina according to the manufacturer’s instructions. After adaptor ligation, RNA samples were subjected to a column-based RiboRid reaction with dideoxy-modified ArOP and without DNase I treatment. RNA purification steps were performed using the RNA Clean & Concentrator Kit for RNA size > 17 nt. The dideoxy-modified ArOPs were prepared by incubating 5.4 nmol (50 pmol each probes) of ArOPs at 37°C for 4 h with 50 U terminal deoxynucleotide transferase (TdT; Thermo), 20 μL 5× Reaction Buffer, 20 mM ddNTP (as a mixture of 5 mM each ddATP, ddTTP, ddGTP, and ddCTP) in 100 μL reaction mixture. The modified ArOP was purified using the Oligo Clean & Concentrator Kit (Zymo) as described by the manufacturer. Then, 3′ RNA adaptor ligation, reverse transcription, and sequencing library amplification were performed using the NEBNext Small RNA Library Prep Set for Illumina. The library was sequenced using an Illumina platform. Information on NGS (run recipe and instrument) is summarized in **S6 Table**.

### Data processing

Sequencing data were processed on a CLC Genomics Workbench (CLC Bio, Aarhus, Denmark). Raw reads were trimmed using the Trim Sequence Tool in NGS Core Tools with a quality limit of 0.05. Reads with more than two ambiguous nucleotides were discarded and the quality trimmed reads were mapped on either the reference genome or rRNAs using the following parameters: mismatch cost of 2, indel cost of 3, and similarity and length fraction of 0.9. Reference genome sequences were downloaded from NCBI under the accession numbers NC_000913.3 (*E*. *coli*), NC_016612 (*K*. *oxytoca*), NC_002516.2 (*P*. *aeruginosa*), NC_010079 (*S*. *aureus* DNA), NC_010063 (*S*. *aureus* plasmid pUSA300HOUMR), NC_012417 (*S*. *aureus* plasmid pUSA01-HOU), NC_004663 (*B*. *thetaiotaomicron* chromosomal DNA), NC_004703.1 (*B*. *thetaiotaomicron* plasmid DNA), NZ_CP019962.1 (*E*. *limosum*), and NC_003888.3 (*S*. *coelicolor*). Gene expression levels were calculated by counting strand-specific reads of genes (antisense) and normalized using DESeq2 [20]. Nonspecific ArOP binding was predicted by standalone BLASTN alignments of probes to genomic sequences lacking rRNA sequences. For motif analysis, DNA sequences 40 nt upstream to 20 nt downstream of the 3′ ends of the transcript were analyzed using MEME software (v5.0.4) using the mode of zero or one site per sequence [35]. The found motif had an E-value of 4.2 × 10^−55^ and each sequence aligned to the motif with a *p*-value lower than 1.32 × 10^−4^. The meta-analysis of ribosome profiles was performed using the STATR pipeline, as described previously [36]. Wilcoxon’s rank-sum test was performed via statistical analysis using the SciPy package [37]

## Supporting information

S1 Protocol Column-based RiboRid. (PDF)

S2 Protocol C3-spacer modified ArOP and SPRI-based RiboRid. (PDF)

S1 Fig Electrophoretic size analysis of RNA samples before and after treatment of RiboRid. (A) Total RNA sample used. Bands of 16S and 23S rRNA are annotated. (B) RNA sample after RiboRid treatment. There was no observable rRNA and significant degradation of mRNA.
(PNG)

S2 Fig Profiles of RNA-Seq conducted from RNA samples prepared with different rRNA removal method. RNA-Seq profiles on (A) *rpsA* gene and (B) *pdhR*-*aceEF*-*lpd* operon. (C) Pairwise comparison of cumulative read count of RNA-Seq profiles on *rpsA* gene.
(PNG)

S3 Fig Read length distributions of RNA-Seq performed from RNA samples prepared by different rRNA removal method. Read length distributions of (A) rRNA reads and (B) non-rRNA reads detected by RNA-Seq of RNA samples without rRNA removal or treated with MICROB*Express*. Read length distributions of (C) rRNA reads and (D) non-rRNA reads detected by RNA-Seq of RNA samples prepared by Ribo-Zero, RiboErase, or RiboRid.
(PNG)

S4 Fig Reproducibility of RNA-Seq library constructed from RNAs prepared using one of the three different rRNA depletion methods. (A) Pairwise Pearson’s correlations (R^2^) between samples and biological replicates. (B) Scatter plots showing mRNA expression level measured by RNA-Seq from different rRNA depletion methods. Each circles indicate individual genes.
(PNG)

S5 Fig Mean-standard deviation plot of RNA-Seq samples prepared by different rRNA removal methods. Mean and standard deviation are calculated from log2 transformed pseudo-count (Log_2_(expression level+1)). Rank indicates rank of mean expression in an ascending order (the higher the rank is, the higher the mean expression is). The orange lines show moving averages of standard deviation with a window size of 50 genes. n: number of genes detected. Average CV: average coefficient of variation (standard deviation divided by mean expression of a gene) of detected genes.
(PNG)

S6 Fig Reproducibility of RNA-Seq library constructed from RNAs prepared using different experimental conditions of RiboRid. (A) Reproducibility of RNA-Seq with different combination of input RNA, amount of ArOP used, and reaction temperature of performing RiboRid. (B) Pairwise comparison between gene expression levels measured by two different technical replicates of RNA-Seq results. (C) Reproducibility of RNA-Seq prepared from the standard RiboRid method using column or the method with C3 spacer-modified ArOPs and SPRI bead-based purification.
(PNG)

S7 Fig Pairwise comparison of gene expression levels measured by RNA-Seq performed from RNA samples with different treatments. Genes with different degree of sequence similarity (*E*-value; BLASTN) were compared as a group. E<1; ≥15 nt: genes with *E*-value lower than 1 (BLASTN alignment with ArOP) and with 15 nt or more consecutive matches. ns: difference between fold-change distributions are not significant (Wilcoxon’s rank-sum test). Box limits, whiskers, and center lines indicate 1^st^ and 3^rd^ quartiles, 10^th^ and 90^th^ percentiles, and the median of the distribution, respectively. Dots are individual genes. Number of subjected genes are indicated above the graph. ns: not significant (Wilcoxon’s rank-sum test).
(PNG)

S8 Fig Reproducibility of RNA-Seq of *P*. *aeruginosa*, *S*. *aureus*, and *B*. *thetaiotaomicron*. Pairwise Pearson’s correlation (R^2^) between biological replicates of (A) *P*. *aeruginosa*, (B) *S*. *aureus*, and (C) *B*. *thetaiotaomicron*.
(PNG)

S9 Fig Reproducibility of two biological replicates of Term-Seq. Term-Seq profiles on genes encoding ribosomal protein (A) RplT and (B) RpmJ. The two biological replicates correlate to each other with a single nucleotide precision without any noticeable noise. (C) Pairwise comparison of Term-Seq signal from the two biological replicates showed linear correlation (Pearson’s R^2^ of 0.9998) throughout the genome.
(PNG)

S10 Fig Meta-analysis of ribosome profile of Ribo-Zero-treated sample aligned at the start codon by different read assignment method. Either 5′ or 3′ ends of sequencing reads were used to determine boundary of ribosome. Ribosome density (RD) is average ribosome profile of coding sequences normalized by dividing ribosome profile of each positions with the maximum peak height in 400 nt window.
(PNG)

S1 Table Sequencing methods used in this study. SE: single-ended recipe. PE: pair-ended recipe.
(XLSX)

S2 Table Anti-rRNA oligonucleotide probes for *E*. *coli*. (XLSX)

S3 Table Predicted binding of *E*. *coli* anti-rRNA oligonucleotide probes on mRNA. (XLSX)

S4 Table C3-spacer modified anti-rRNA oligonucleotide probes for *E*. *coli*. 3SpC3: C3-spacer modification at the 3′ end.
(XLSX)

S5 Table Anti-rRNA oligonucleotide probes used in this study for various bacterial species. (XLSX)

S6 Table Additional ArOP targeting enriched regions of ribosomal RNA in ribosome profiling. (XLSX)

S7 Table Numerical data supports figures. (XLSX)

## References

[1] Schena M, Shalon D, Davis RW, Brown PO. Quantitative monitoring of gene expression patterns with a complementary DNA microarray. Science. 1995;270(5235):467–470. doi: 10.1126/science.270.5235.467 7569999

[2] Nagalakshmi U, Wang Z, Waern K, Shou C, Raha D, Gerstein M, et al. The transcriptional landscape of the yeast genome defined by RNA sequencing. Science. 2008;320(5881):1344–1349. doi: 10.1126/science.1158441 18451266 PMC2951732

[3] Morlan JD, Qu K, Sinicropi DV. Selective depletion of rRNA enables whole transcriptome profiling of archival fixed tissue. PLoS One. 2012;7(8):e42882. doi: 10.1371/journal.pone.0042882 22900061 PMC3416766

[4] Zhao J, Hyman L, Moore C. Formation of mRNA 3’ ends in eukaryotes: mechanism, regulation, and interrelationships with other steps in mRNA synthesis. Microbiol Mol Biol Rev. 1999;63(2):405–445. doi: 10.1128/MMBR.63.2.405-445.1999 10357856 PMC98971

[5] Su C, Sordillo LM. A simple method to enrich mRNA from total prokaryotic RNA. Mol Biotechnol. 1998;10(1):83–85. doi: 10.1007/BF02745865 9779425

[6] Pang X, Zhou D, Song Y, Pei D, Wang J, Guo Z, et al. Bacterial mRNA purification by magnetic capture-hybridization method. Microbiol Immunol. 2004;48(2):91–96. doi: 10.1111/j.1348-0421.2004.tb03493.x 14978333

[7] Culviner PH, Guegler CK, Laub MT. A simple, cost-effective, and robust method for rRNA depletion in RNA-sequencing studies. mBio. 2020;11(2). 10.1128/mBio.00010-20 PMC7175087 32317317

[8] Kang Y, Norris MH, Zarzycki-Siek J, Nierman WC, Donachie SP, Hoang TT. Transcript amplification from single bacterium for transcriptome analysis. Genome Res. 2011;21(6):925–935. doi: 10.1101/gr.116103.110 21536723 PMC3106325

[9] Zhulidov PA, Bogdanova EA, Shcheglov AS, Vagner LL, Khaspekov GL, Kozhemyako VB, et al. Simple cDNA normalization using kamchatka crab duplex-specific nuclease. Nucleic Acids Res. 2004;32(3):e37. doi: 10.1093/nar/gnh031 14973331 PMC373426

[10] Yi H, Cho YJ, Won S, Lee JE, Jin Yu H, Kim S, et al. Duplex-specific nuclease efficiently removes rRNA for prokaryotic RNA-seq. Nucleic Acids Res. 2011;39(20):e140. doi: 10.1093/nar/gkr617 21880599 PMC3203590

[11] McGrath KC, Thomas-Hall SR, Cheng CT, Leo L, Alexa A, Schmidt S, et al. Isolation and analysis of mRNA from environmental microbial communities. J Microbiol Methods. 2008;75(2):172–176. doi: 10.1016/j.mimet.2008.05.019 18582973

[12] Wangsanuwat C, Heom KA, Liu E, O’Malley MA, Dey SS. Efficient and cost-effective bacterial mRNA sequencing from low input samples through ribosomal RNA depletion. BMC Genomics. 2020;21(1):717. doi: 10.1186/s12864-020-07134-4 33066726 PMC7565789

[13] Huang Y, Sheth RU, Kaufman A, Wang HH. Scalable and cost-effective ribonuclease-based rRNA depletion for transcriptomics. Nucleic Acids Res. 2020;48(4):e20. doi: 10.1093/nar/gkz1169 31879761 PMC7038938

[14] AbouHaidar MG, Ivanov IG. Non-enzymatic RNA hydrolysis promoted by the combined catalytic activity of buffers and magnesium ions. Z Naturforsch C J Biosci. 1999;54(7–8):542–548. doi: 10.1515/znc-1999-7-813 10488562

[15] Petrova OE, Garcia-Alcalde F, Zampaloni C, Sauer K. Comparative evaluation of rRNA depletion procedures for the improved analysis of bacterial biofilm and mixed pathogen culture transcriptomes. Sci Rep. 2017;7:41114. doi: 10.1038/srep41114 28117413 PMC5259769

[16] Giannoukos G, Ciulla DM, Huang K, Haas BJ, Izard J, Levin JZ, et al. Efficient and robust RNA-seq process for cultured bacteria and complex community transcriptomes. Genome Biol. 2012;13(3):R23. doi: 10.1186/gb-2012-13-3-r23 22455878 PMC3439974

[17] Prezza G, Heckel T, Dietrich S, Homberger C, Westermann AJ, Vogel J. Improved bacterial RNA-seq by Cas9-based depletion of ribosomal RNA reads. RNA. 2020;26(8):1069–1078. doi: 10.1261/rna.075945.120 32345633 PMC7373992

[18] Zinshteyn B, Wangen JR, Hua B, Green R. Nuclease-mediated depletion biases in ribosome footprint profiling libraries. RNA. 2020;26(10):1481–1488. doi: 10.1261/rna.075523.120 32503920 PMC7491325

[19] McCarthy DJ, Chen Y, Smyth GK. Differential expression analysis of multifactor RNA-Seq experiments with respect to biological variation. Nucleic Acids Res. 2012;40(10):4288–4297. doi: 10.1093/nar/gks042 22287627 PMC3378882

[20] Love MI, Huber W, Anders S. Moderated estimation of fold change and dispersion for RNA-seq data with DESeq2. Genome Biol. 2014;15(12):550. doi: 10.1186/s13059-014-0550-8 25516281 PMC4302049

[21] Gales AC, Jones RN, Turnidge J, Rennie R, Ramphal R. Characterization of *Pseudomonas aeruginosa* isolates: occurrence rates, antimicrobial susceptibility patterns, and molecular typing in the global SENTRY Antimicrobial Surveillance Program, 1997–1999. Clin Infect Dis. 2001;32 Suppl 2:S146–155. 11320454 10.1086/320186

[22] Stover CK, Pham XQ, Erwin AL, Mizoguchi SD, Warrener P, Hickey MJ, et al. Complete genome sequence of *Pseudomonas aeruginosa* PAO1, an opportunistic pathogen. Nature. 2000;406(6799):959–964. doi: 10.1038/35023079 10984043

[23] Deresinski S. Methicillin-resistant *Staphylococcus aureus*: an evolutionary, epidemiologic, and therapeutic odyssey. Clin Infect Dis. 2005;40(4):562–573. doi: 10.1086/427701 15712079

[24] Highlander SK, Hulten KG, Qin X, Jiang H, Yerrapragada S, Mason EO Jr., et al. Subtle genetic changes enhance virulence of methicillin resistant and sensitive *Staphylococcus aureus*. BMC Microbiol. 2007;7:99. doi: 10.1186/1471-2180-7-99 17986343 PMC2222628

[25] Song Y, Cho BK. Draft genome sequence of chemolithoautotrophic acetogenic butanol-producing *Eubacterium limosum* ATCC 8486. Genome Announc. 2015;3(1). doi: 10.1128/genomeA.01564-14 25676768 PMC4333668

[26] Faith JJ, Guruge JL, Charbonneau M, Subramanian S, Seedorf H, Goodman AL, et al. The long-term stability of the human gut microbiota. Science. 2013;341(6141):1237439. doi: 10.1126/science.1237439 23828941 PMC3791589

[27] Wexler AG, Goodman AL. An insider’s perspective: *Bacteroides* as a window into the microbiome. Nat Microbiol. 2017;2:17026. doi: 10.1038/nmicrobiol.2017.26 28440278 PMC5679392

[28] Dar D, Shamir M, Mellin JR, Koutero M, Stern-Ginossar N, Cossart P, et al. Term-seq reveals abundant ribo-regulation of antibiotics resistance in bacteria. Science. 2016;352(6282):aad9822. doi: 10.1126/science.aad9822 27120414 PMC5756622

[29] Lee Y, Lee N, Hwang S, Kim W, Jeong Y, Cho S, et al. Genome-scale determination of 5′ and 3′ boundaries of RNA transcripts in *Streptomyces* genomes. Sci Data. 2020;7(1):436. doi: 10.1038/s41597-020-00775-w 33319794 PMC7738537

[30] Lee Y, Lee N, Jeong Y, Hwang S, Kim W, Cho S, et al. The transcription unit architecture of *Streptomyces lividans* TK24. Front Microbiol. 2019;10:2074. doi: 10.3389/fmicb.2019.02074 31555254 PMC6742748

[31] Ingolia NT, Ghaemmaghami S, Newman JR, Weissman JS. Genome-wide analysis *in vivo* of translation with nucleotide resolution using ribosome profiling. Science. 2009;324(5924):218–223. doi: 10.1126/science.1168978 19213877 PMC2746483

[32] Latif H, Szubin R, Tan J, Brunk E, Lechner A, Zengler K, et al. A streamlined ribosome profiling protocol for the characterization of microorganisms. BioTechniques. 2015;58(6):329–332. doi: 10.2144/000114302 26054770

[33] Woolstenhulme CJ, Guydosh NR, Green R, Buskirk AR. High-precision analysis of translational pausing by ribosome profiling in bacteria lacking EFP. Cell Rep. 2015;11(1):13–21. doi: 10.1016/j.celrep.2015.03.014 25843707 PMC4835038

[34] Stead MB, Agrawal A, Bowden KE, Nasir R, Mohanty BK, Meagher RB, et al. RNAsnap: a rapid, quantitative and inexpensive, method for isolating total RNA from bacteria. Nucleic Acids Res. 2012;40(20):e156. doi: 10.1093/nar/gks680 22821568 PMC3488207

[35] Bailey TL, Boden M, Buske FA, Frith M, Grant CE, Clementi L, et al. MEME SUITE: tools for motif discovery and searching. Nucleic Acids Res. 2009;37(Web Server issue):W202–208. doi: 10.1093/nar/gkp335 19458158 PMC2703892

[36] Choe D, Palsson B, Cho BK. STATR: A simple analysis pipeline of Ribo-Seq in bacteria. J Microbiol. 2020;58(3):217–226. doi: 10.1007/s12275-020-9536-2 31989542 PMC7209825

[37] Oliphant TE. Python for scientific computing. Comput Sci Eng. 2007;9:10–20.

