# Supplementary material for: RiboRid: A low cost, advanced, and ultra-efficient method to remove ribosomal RNA for bacterial transcriptomics
Source: PLoS Genet. 2021 Sep 27;17(9):e1009821. doi: 10.1371/journal.pgen.1009821 (PMC8496792; doi:10.1371/journal.pgen.1009821)
Supplement: S1 Protocol — (PDF) [file pgen.1009821.s001.pdf]

S1 Protocol. Column-based RiboRid

Materials

Material	Ref. Num.	Required amount/rxn	Cost/rxn (unit cost)
ArOP	IDT	5 pmoles each probes	$0.387 ($774.5 for synthesis, enough for 2,000 rxn)
DNase I	M0303S (NEB)	12 U	$0.664 ($55.4, enough for 83 rxn)
RNA Clean and Concentrator Kit	R1013 (Zymo)	1 column	$2.366 ($118.3, enough for 50 rxn)
Hybridase™ Thermostable RNase H	H39500 (Lucigen)	10 U	$8.460 ($423, enough for 50 rxn)
**Total**			**$11.88**

Thermocycler programs

Thermocycler Program 1. Hybridase Reaction

Step	Temperature	Duration	Reaction volume	Description
1	37°C	Hold	15 µl	
2	37°C	10 min	15 µl	DNase I reaction
3	25°C	Hold	15 µl	Addition of Hybridase Complement Buffer
4	75°C	10 min	30 µl	Inactivation of DNase I
5	25°C	Hold	30 µl	Addition of ArOP and $MgCl_2$
6	90°C	1 sec	32 µl	Denaturation of RNA
7	65°C	Hold	32 µl	Addition of Hybridase
8	65°C	20 min	34 µl	Ribosomal RNA depletion cycle 1
9	90°C	1 sec	34 µl	Denaturation of RNA, recycling ArOP
10	65°C	10 min	34 µl	Ribosomal RNA depletion cycle 2
11	65°C	Hold	34 µl	

Thermocycler Program 2. ArOP Depletion Reaction

Temperature	Duration	Reaction volume
25°C	5 min	50 µl
Ramp to 30°C with a rate of 0.1°C/sec		
30°C	5 min	50 µl
Ramp to 35°C with a rate of 0.1°C/sec		
35°C	5 min	50 µl
Ramp to 40°C with a rate of 0.1°C/sec		
40°C	5 min	50 µl
Ramp to 45°C with a rate of 0.1°C/sec		
45°C	5 min	50 µl
25°C	Hold	50 µl

Protocol

1) Preheat thermocycler at 37°C by starting **Thermocycler Program 1**.
2) Mix the following in a 0.2 ml-thin-walled tube:

Component	Amount
Total RNA sample	0.5-1 µg*

10× DNase I Buffer (NEB)	1.5 µl
Nuclease-free DNase I (NEB)	1 µl (2 U)
Nuclease-free water	up to 15 µl
**Total**	**15 µl**

*The amount of RNA was measured fluorometrically using Qubit HS RNA.

3) Place the mixture in the preheated thermocycler and proceed to Step 2 of **Thermocycler Program 1**.
4) When the sample reaches 25°C C (Step 3 of **Thermocycler Program 1**), remove the tube from the thermocycler.
5) Add 15 µl of **Hybridase Complement Buffer** and mix well by pipetting.

Hybridase Complement Buffer

Component	Concentration	Amount
Tris-HCl (pH 7.5)	1 M	90 µl
KCl	1 M	200 µl
Nuclease-free water	-	710 µl
**Total**		**1 ml**

6) Proceed to Step 4 of **Thermocycler Program 1**.
7) When the sample reaches 25°C (Step 5 of **Thermocycler Program 1**), remove the tube from the thermocycler.
8) Add 1 µl (540 pmoles, 5 pmoles each) of ArOP mix and 1 µl of 100 mM RNase-free $MgCl_2$ to the sample.
9) Mix well by pipetting and return the sample to thermocycler.
10) Proceed to Step 6 of **Thermocycler Program 1**.
11) When the sample reaches 65°C (Step 7 of **Thermocycler Program 1**), open the tube lid and add 2 µl of Hybridase (pre-warmed to room temperature) without removing the sample from thermocycler.
12) Mix the sample by pipetting entire reaction carefully without removing the sample from thermocycler.
13) Close the tube lid and proceed to Step 8 of **Thermocycler Program 1**.
14) When the sample reaches 65°C (Step 11 of **Thermocycler Program 1**), open the tube lid and add 100 µl of RNA Binding Solution (RNA Clean and Concentrator Kit) without removing the sample from thermocycler.
15) Mix well by pipetting and remove the sample from thermocycler.
16) Add 66 µl of nuclease-free water and 100 µl of absolute ethanol to the sample.
17) Apply the mixture to a Zymo-Spin IC Column (RNA Clean and Concentrator Kit) and centrifuge at 8,000 ×g for 30 sec.
18) Discard flow-through and apply 400 µl of RNA Prep Buffer.
19) Centrifuge at 8,000 ×g for 30 sec.
20) Discard flow-through and apply 600 µl of RNA Wash Buffer.
21) Centrifuge at 8,000 ×g for 30 sec.
22) Discard flow-through and apply 400 µl of RNA Wash Buffer.
23) Centrifuge at 16,000 ×g for 2 min.
24) Discard flow-through and apply 40 µl of nuclease-free water to the column matrix.
25) Centrifuge at 16,000 ×g for 30 sec.
26) Elute the column again by re-applying the flow-through to the column matrix and centrifuging at 16,000 ×g for 30 sec. Save the column for re-use in Step 31.
27) Mix the following in a 0.2 ml-thin-walled tube:

Component	Amount
rRNA-depleted RNA sample	40 µl
10× DNase I Buffer (NEB)	5 µl
Nuclease-free DNase I (NEB)	5 µl (10 U)
**Total**	**50 µl**

28) Place the tube in thermocycler and start **Thermocycler Program 2**.
29) After incubation, transfer the sample to a new 1.5 ml microcentrifuge tube containing mixture of 100 µl of RNA Binding Solution and 50 µl of nuclease-free water. Vortex vigorously.
30) Add 100 µl of absolute ethanol.
31) Repeat Step 17-25).
32) Elute RNA with 10 µl of nuclease-free water for two times. Typically, 50-100 ng (5-10%) of rRNA-removed RNA is recovered from 500-1000 ng of input RNA.
