# Supplementary material for: RiboRid: A low cost, advanced, and ultra-efficient method to remove ribosomal RNA for bacterial transcriptomics
Source: PLoS Genet. 2021 Sep 27;17(9):e1009821. doi: 10.1371/journal.pgen.1009821 (PMC8496792; doi:10.1371/journal.pgen.1009821)
Supplement: S2 Protocol — (PDF) [file pgen.1009821.s002.pdf]

S2 Protocol. C3-spacer modified ArOP and SPRI-based RiboRid

Materials

Material	Ref. Num.	Required amount/rxn	Cost/rxn (unit cost)
C3-modified ArOP	IDT	5 pmoles each probes	$0.506 ($3,038 for synthesis, enough for 6,000 rxn)
DNase I	M0303S (NEB)	2 U	$0.111 ($55.4, enough for 500 rxn)
CleanNGS DNA & RNA SPRI Bead Purification Kit	CNGS050 (Bulldog Bio)	88 µl	$1.047 ($595, enough for 568 rxn)
Hybridase^{TM} Thermostable RNase H	H39500 (Lucigen)	10 U	$8.460 ($423, enough for 50 rxn)
**Total**			**$10.12**

Thermocycler program

Thermocycler Program 1. Hybridase Reaction

Step	Temperature	Duration	Reaction volume	Description
1	37°C	Hold	15 µl	
2	37°C	10 min	15 µl	DNase I reaction
3	25°C	Hold	15 µl	Addition of Hybridase Complement Buffer
4	75°C	10 min	30 µl	Inactivation of DNase I
5	25°C	Hold	30 µl	Addition of ArOP and $MgCl_2$
6	90°C	1 sec	32 µl	Denaturation of RNA
7	65°C	Hold	32 µl	Addition of Hybridase
8	65°C	20 min	34 µl	Ribosomal RNA depletion cycle 1
9	90°C	1 sec	34 µl	Denaturation of RNA, recycling ArOP
10	65°C	10 min	34 µl	Ribosomal RNA depletion cycle 2
11	65°C	Hold	34 µl	

Protocol

1) Preheat thermocycler at 37°C by starting **Thermocycler Program 1**.
2) Mix the following in a 0.2 ml-thin-walled tube:

Component	Amount
Total RNA sample	0.5-1 µg*
10× DNase I Buffer (NEB)	1.5 µl
Nuclease-free DNase I (NEB)	1 µl (2 U)
Nuclease-free water	up to 15 µl
**Total**	**15 µl**

*The amount of RNA was measured fluorometrically using Qubit HS RNA.

3) Place the mixture in the preheated thermocycler and proceed to Step 2 of **Thermocycler Program 1**.
4) When the sample reaches 25°C C (Step 3 of **Thermocycler Program 1**), remove the tube from the thermocycler.
5) Add 15 µl of **Hybridase Complement Buffer** and mix well by pipetting.

Hybridase Complement Buffer

Component	Concentration	Amount
Tris-HCl (pH 7.5)	1 M	90 µl
KCl	1 M	200 µl
Nuclease-free water	-	710 µl
**Total**		**1 ml**

6) Proceed to Step 4 of **Thermocycler Program 1**.
7) When the sample reaches 25°C (Step 5 of **Thermocycler Program 1**), remove the tube from the thermocycler.
8) Add 1 µl (540 pmoles, 5 pmoles each) of ArOP mix and 1 µl of 100 mM RNase-free $MgCl_2$ to the sample.
9) Mix well by pipetting and return the sample to thermocycler.
10) Proceed to Step 6 of **Thermocycler Program 1**.
11) When the sample reaches 65°C (Step 7 of **Thermocycler Program 1**), open the tube lid and add 2 µl of Hybridase (pre-warmed to room temperature) without removing the sample from thermocycler.
12) Mix the sample by pipetting entire reaction carefully without removing the sample from thermocycler.
13) Close the tube lid and proceed to Step 8 of **Thermocycler Program 1**.
14) When the sample reaches 65°C (Step 11 of **Thermocycler Program 1**), open the tube lid and add 16.5 µl of 30 mM EDTA solution without removing the sample from thermocycler.
15) Mix well by pipetting and remove the sample from thermocycler.
16) Add 90 µl of SPRI bead solution (homogenize before use) to the sample and mix by vigorous vortexing.
17) Incubate the mixture at room temperature for 5 min.
18) Briefly spin the tube and place it on a magnetic stand.
19) When the solution is clear (takes approximately 2 min), carefully aspirate the supernatant.
20) Add 200 µl of freshly prepared 80% ethanol solution to the tube.
21) Incubate at room temperature for 30 sec.
22) Remove the supernatant.
23) Repeat Step 20-22).
24) Briefly spin the tube and remove residual liquid completely.
25) Air dry beads with cap open for 10 min on the magnetic stand at room temperature.
26) Remove the tube from the magnetic stand and resuspend beads with 15 µl of nuclease-free water.
27) Incubate at room temperature for 2 min.
28) Place the tube on the magnetic stand.
29) After solution is clear, transfer 13 µl of supernatant to a new 1.5-ml microcentrifuge tube. Typically, 50-100 ng (5-10%) of rRNA-removed RNA is recovered from 500-1000 ng of input RNA.
