# Supplementary figures and images for: RiboRid: A low cost, advanced, and ultra-efficient method to remove ribosomal RNA for bacterial transcriptomics
Source: PLoS Genet. 2021 Sep 27;17(9):e1009821. doi: 10.1371/journal.pgen.1009821 (PMC8496792; doi:10.1371/journal.pgen.1009821)

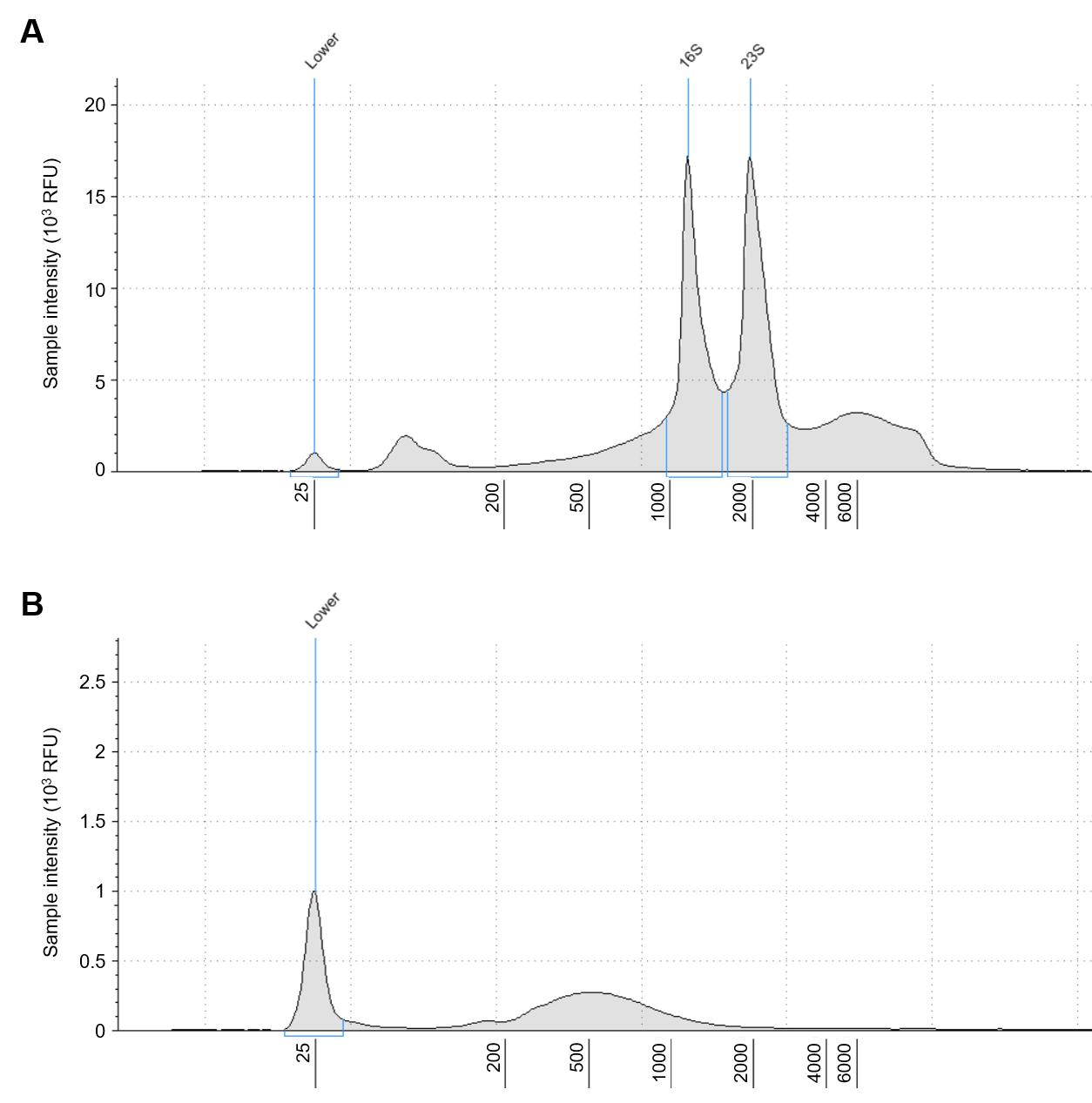

Supplement: S1 Fig — (A) Total RNA sample used. Bands of 16S and 23S rRNA are annotated. (B) RNA sample after RiboRid treatment. There was no observable rRNA and significant degradation of mRNA. (PNG) [file pgen.1009821.s003.png]

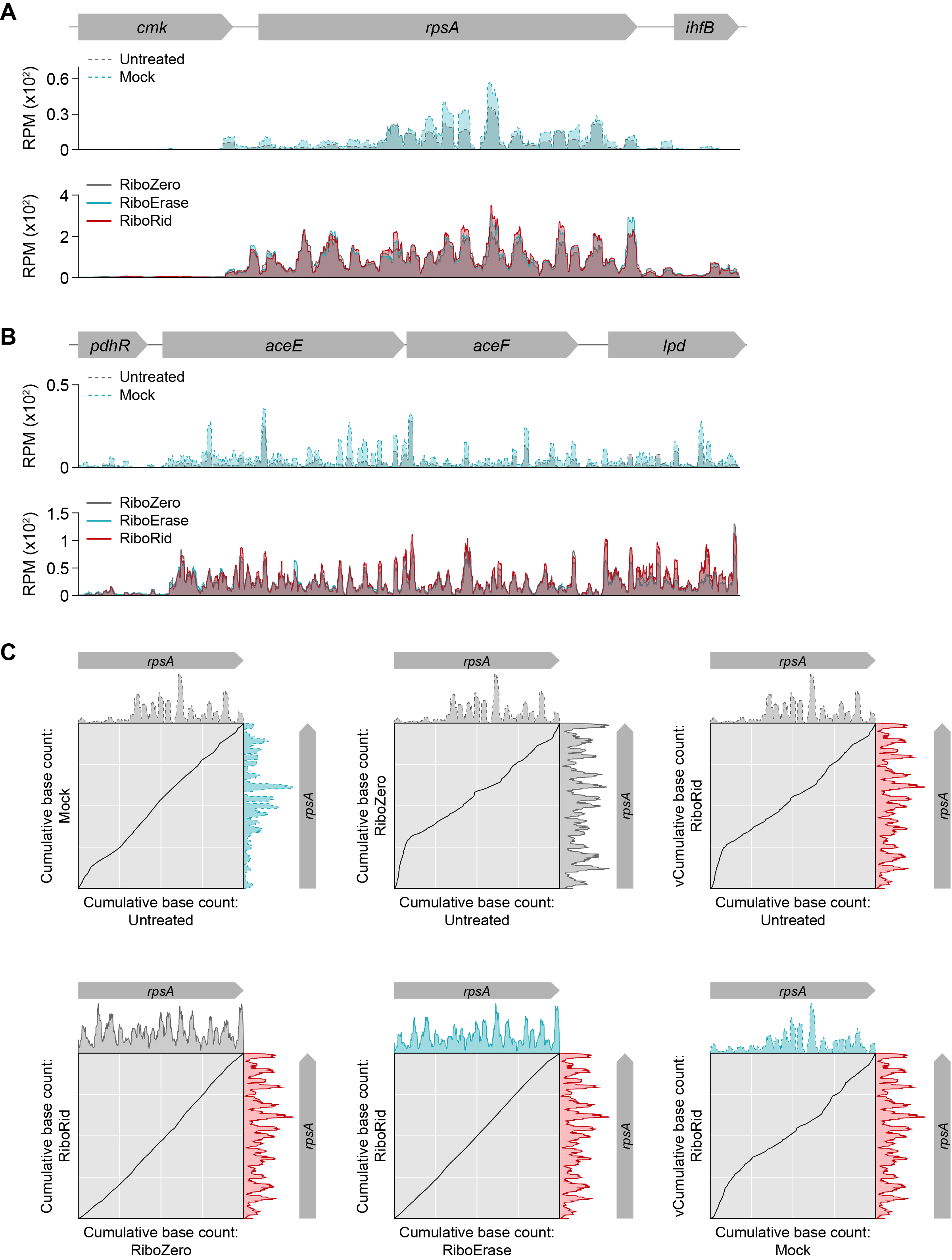

Supplement: S2 Fig — RNA-Seq profiles on (A) rpsA gene and (B) pdhR-aceEF-lpd operon. (C) Pairwise comparison of cumulative read count of RNA-Seq profiles on rpsA gene. (PNG) [file pgen.1009821.s004.png]

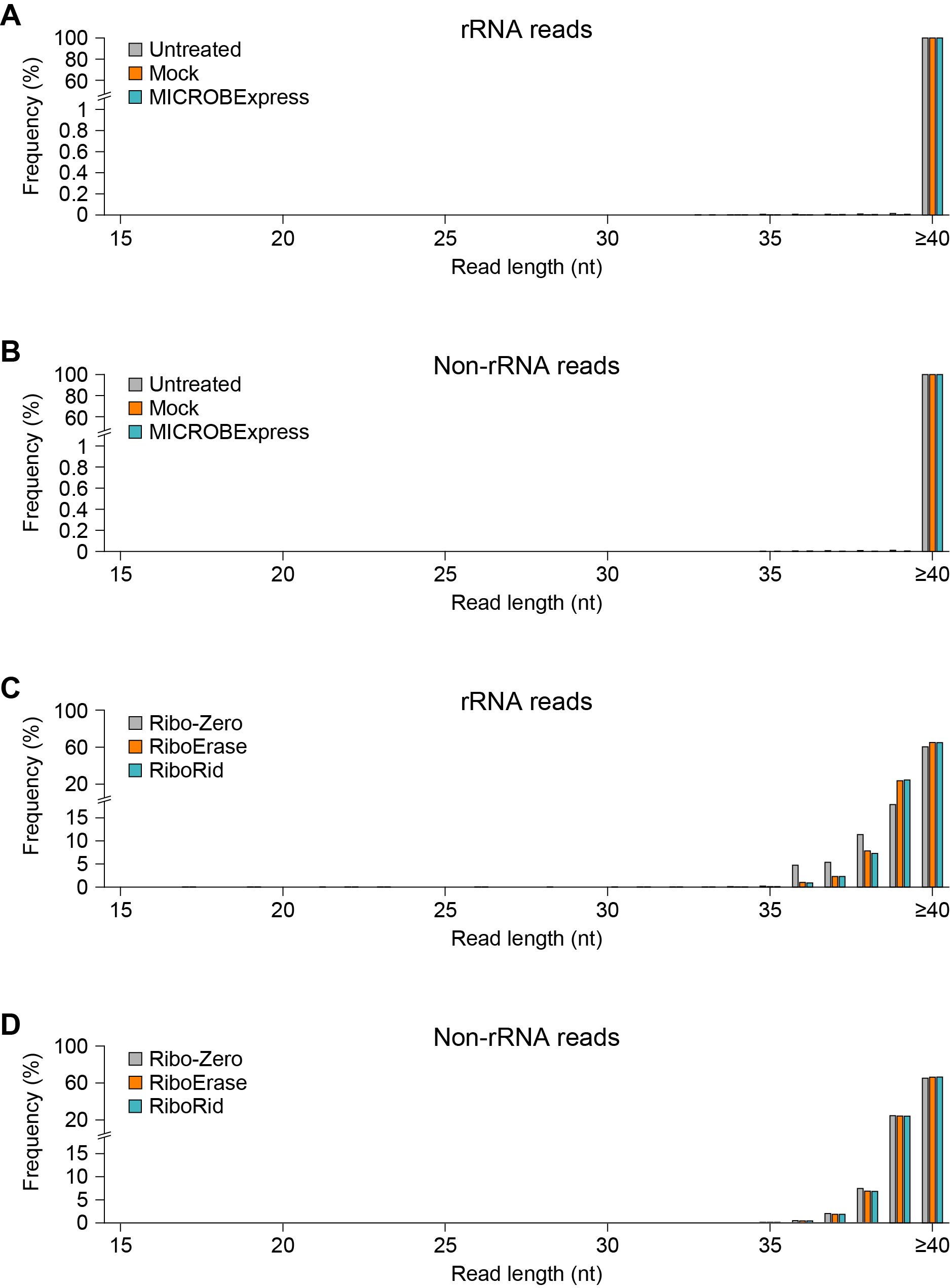

Supplement: S3 Fig — Read length distributions of (A) rRNA reads and (B) non-rRNA reads detected by RNA-Seq of RNA samples without rRNA removal or treated with MICROBExpress. Read length distributions of (C) rRNA reads and (D) non-rRNA reads detected by RNA-Seq of RNA samples prepared by Ribo-Zero, RiboErase, or RiboRid. (PNG) [file pgen.1009821.s005.png]

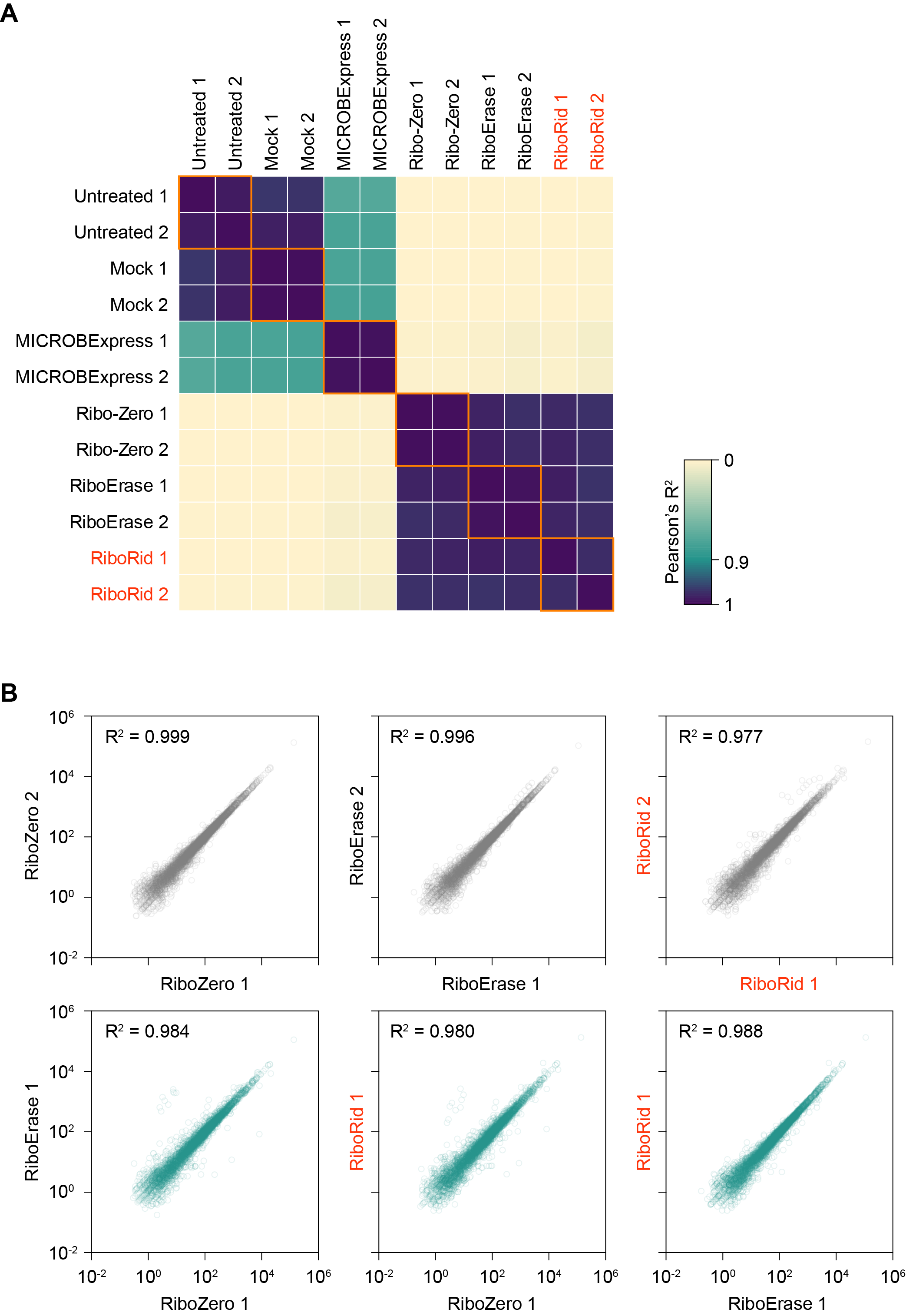

Supplement: S4 Fig — (A) Pairwise Pearson’s correlations (R2) between samples and biological replicates. (B) Scatter plots showing mRNA expression level measured by RNA-Seq from different rRNA depletion methods. Each circles indicate individual genes. (PNG) [file pgen.1009821.s006.png]

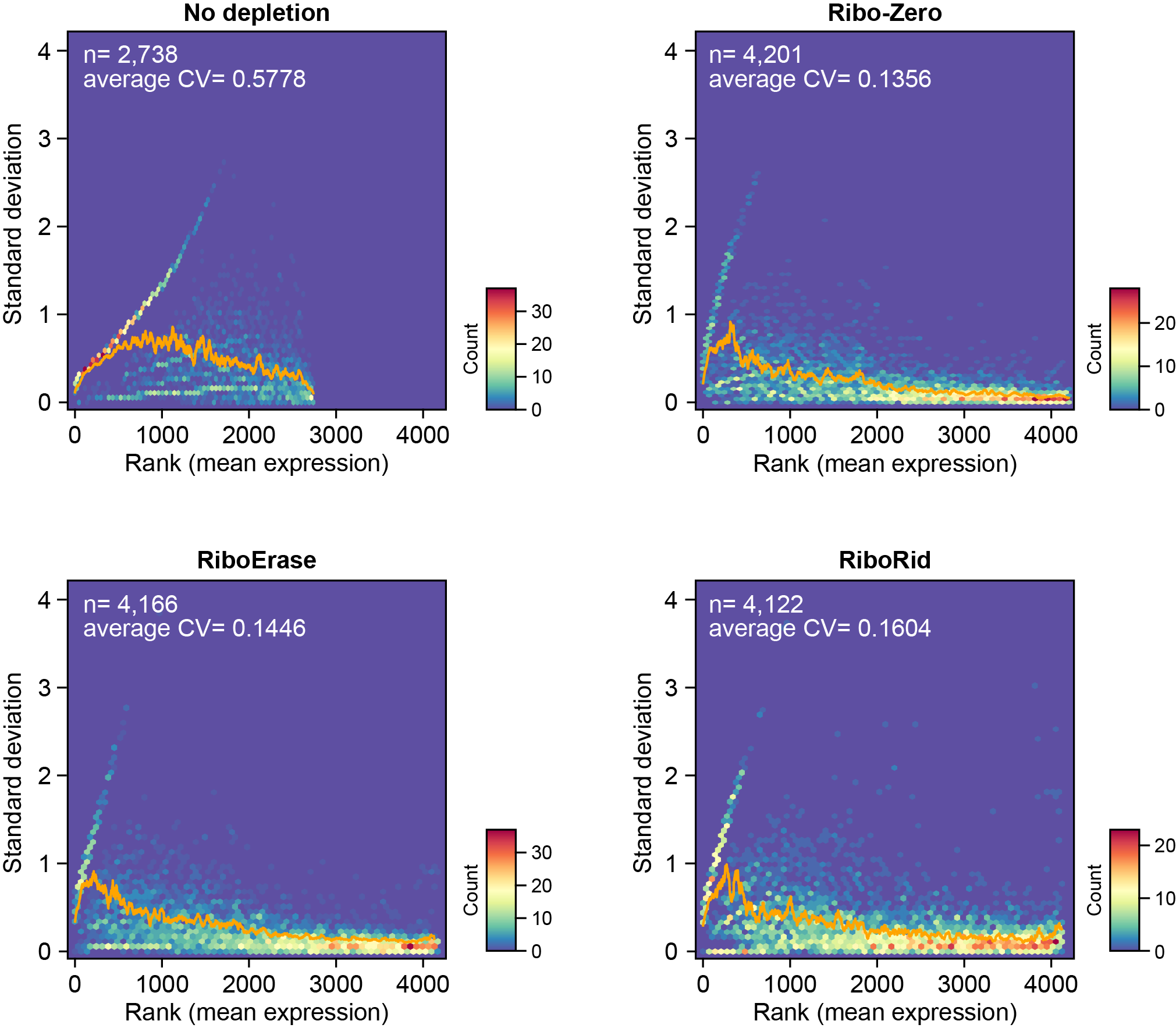

Supplement: S5 Fig — Mean and standard deviation are calculated from log2 transformed pseudo-count (Log2(expression level+1)). Rank indicates rank of mean expression in an ascending order (the higher the rank is, the higher the mean expression is). The orange lines show moving averages of standard deviation with a window size of 50 genes. n: number of genes detected. Average CV: average coefficient of variation (standard deviation divided by mean expression of a gene) of detected genes. (PNG) [file pgen.1009821.s007.png]

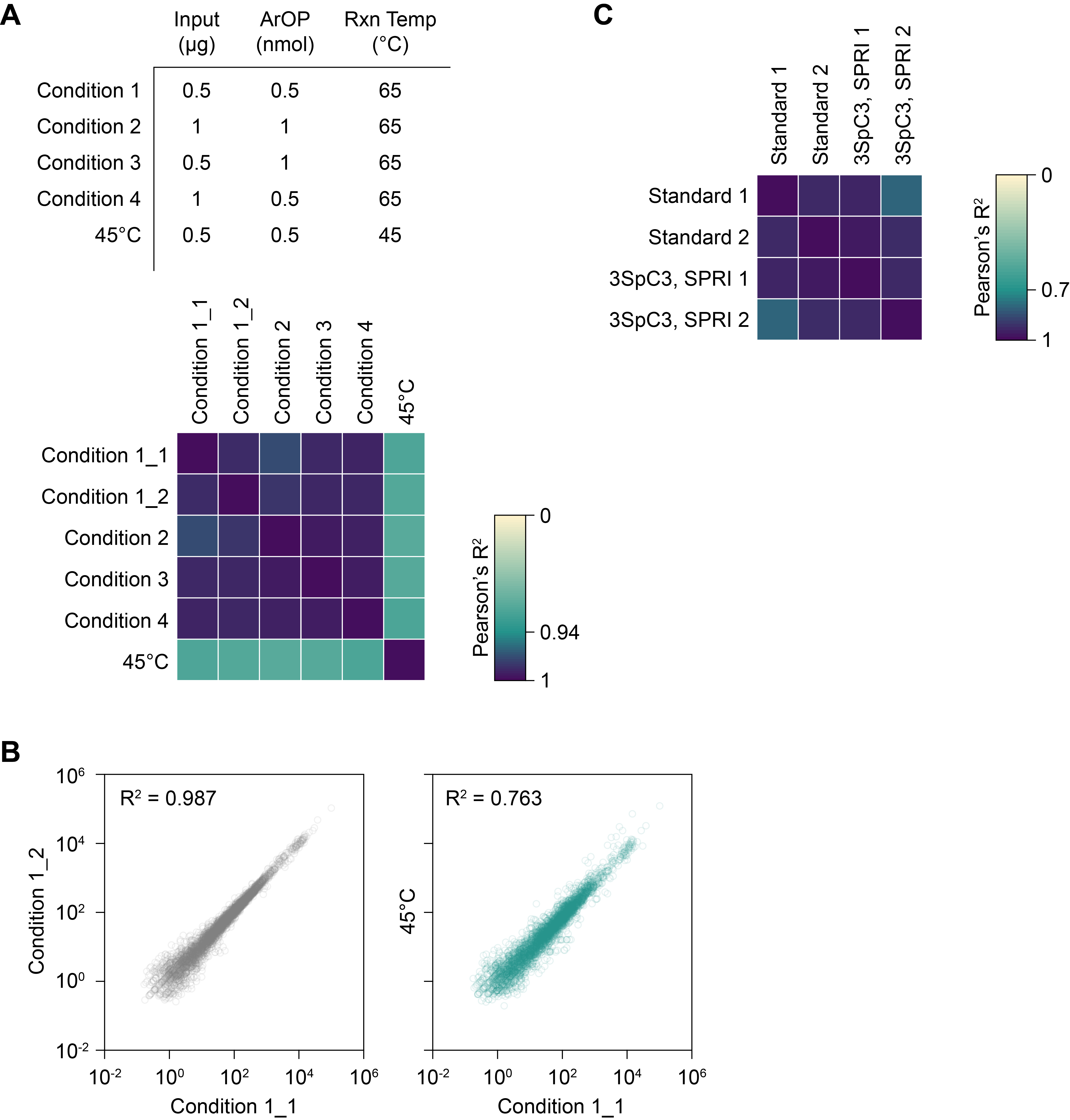

Supplement: S6 Fig — (A) Reproducibility of RNA-Seq with different combination of input RNA, amount of ArOP used, and reaction temperature of performing RiboRid. (B) Pairwise comparison between gene expression levels measured by two different technical replicates of RNA-Seq results. (C) Reproducibility of RNA-Seq prepared from the standard RiboRid method using column or the method with C3 spacer-modified ArOPs and SPRI bead-based purification. (PNG) [file pgen.1009821.s008.png]

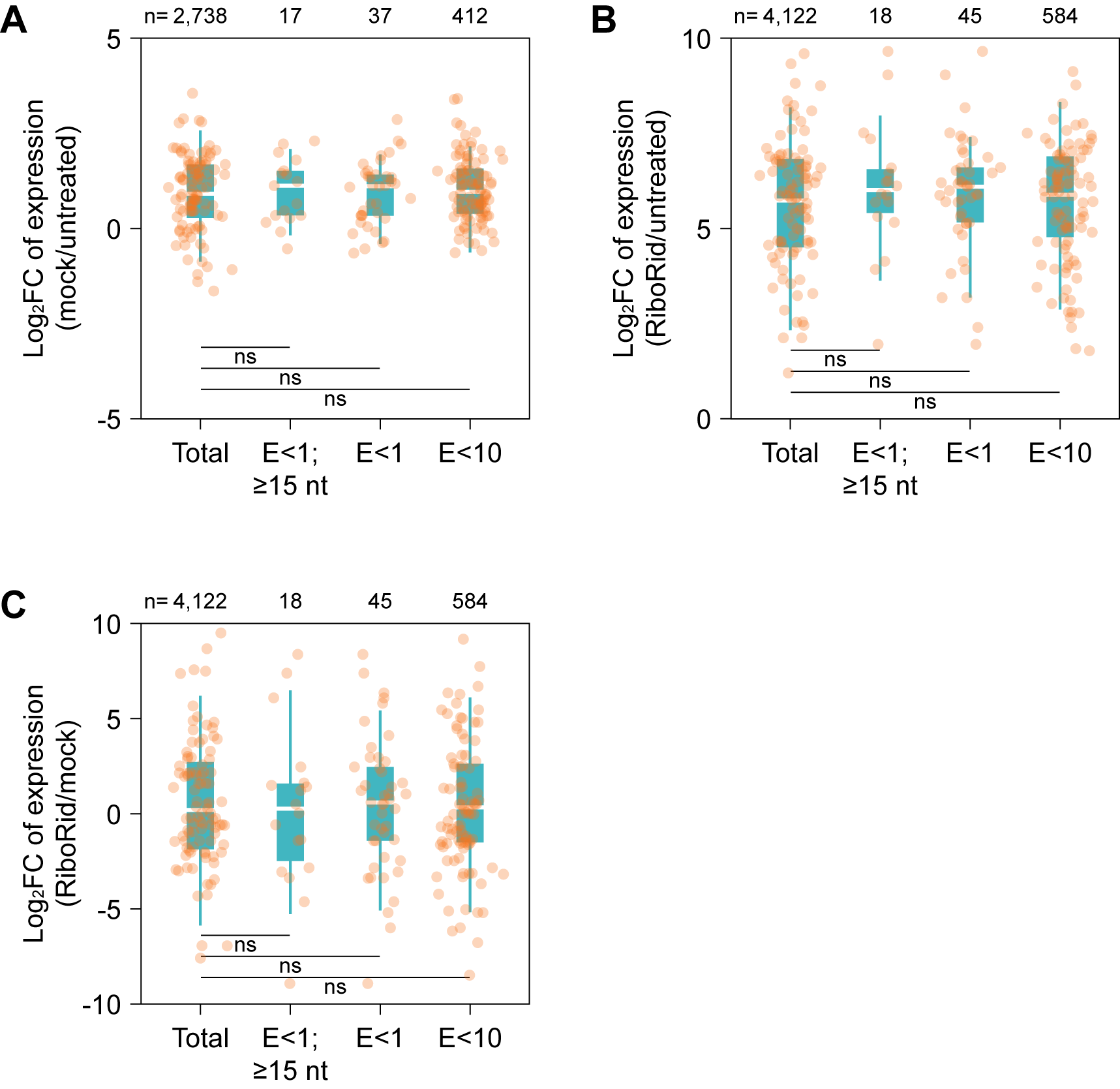

Supplement: S7 Fig — Genes with different degree of sequence similarity (E-value; BLASTN) were compared as a group. E<1; ≥15 nt: genes with E-value lower than 1 (BLASTN alignment with ArOP) and with 15 nt or more consecutive matches. ns: difference between fold-change distributions are not significant (Wilcoxon’s rank-sum test). Box limits, whiskers, and center lines indicate 1st and 3rd quartiles, 10th and 90th percentiles, and the median of the distribution, respectively. Dots are individual genes. Number of subjected genes are indicated above the graph. ns: not significant (Wilcoxon’s rank-sum test). (PNG) [file pgen.1009821.s009.png]

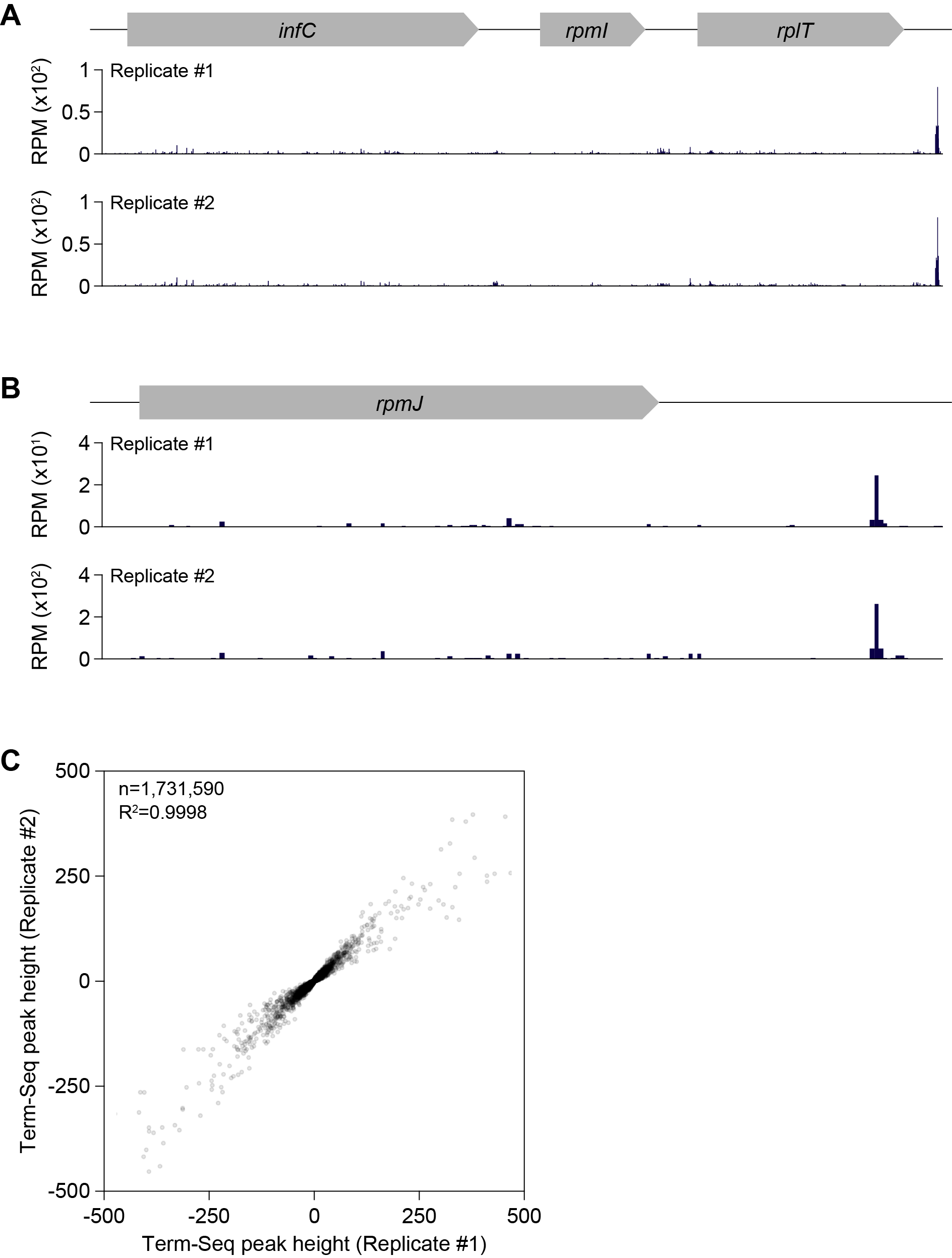

Supplement: S9 Fig — Term-Seq profiles on genes encoding ribosomal protein (A) RplT and (B) RpmJ. The two biological replicates correlate to each other with a single nucleotide precision without any noticeable noise. (C) Pairwise comparison of Term-Seq signal from the two biological replicates showed linear correlation (Pearson’s R2 of 0.9998) throughout the genome. (PNG) [file pgen.1009821.s011.png]

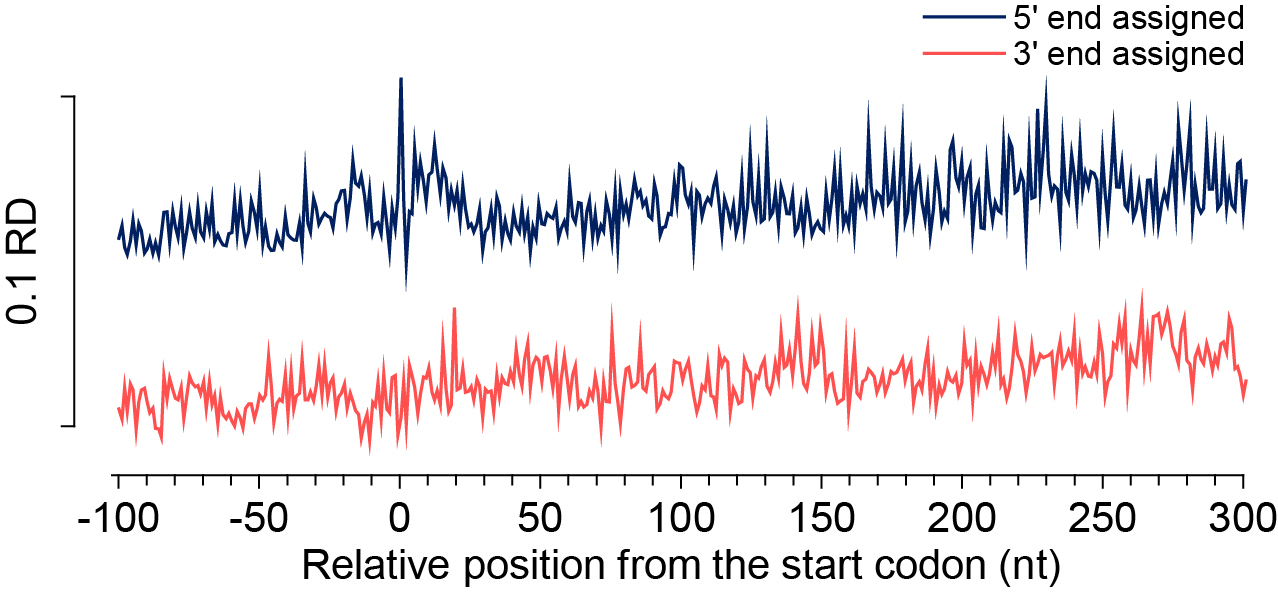

Supplement: S10 Fig — Either 5′ or 3′ ends of sequencing reads were used to determine boundary of ribosome. Ribosome density (RD) is average ribosome profile of coding sequences normalized by dividing ribosome profile of each positions with the maximum peak height in 400 nt window. (PNG) [file pgen.1009821.s012.png]
